# Rapid Discovery and Structure-Property Relationships of Metal-Ion Fluorescent Sensors via Macroarray Synthesis

**DOI:** 10.1038/s41598-019-46783-8

**Published:** 2019-07-17

**Authors:** Apiwat Promchat, Kanet Wongravee, Mongkol Sukwattanasinitt, Thanit Praneenararat

**Affiliations:** 1 Department of Chemistry, Faculty of Science, Chulalongkorn University, Phayathai Rd., Pathumwan, Bangkok 10330 Thailand; 2 Nanotec-CU Center of Excellence on Food and Agriculture, Department of Chemistry, Faculty of Science, Chulalongkorn University, Phayathai Rd., Pathumwan, Bangkok 10330 Thailand; 3 Sensor Research Unit, Department of Chemistry, Faculty of Science, Chulalongkorn University, Phayathai Rd., Pathumwan, Bangkok 10330 Thailand; 4 The Chemical Approaches for Food Applications Research Group, Faculty of Science, Chulalongkorn University, Phayathai Rd., Pathumwan, Bangkok 10330 Thailand

**Keywords:** Fluorescent probes, Sensors, Combinatorial libraries, Solid-phase synthesis, Surface chemistry

## Abstract

A macroarray immobilisation of fluorophores on filter papers for sensing metal ions by *in-situ* reductive amination and carbodiimide coupling is reported herein. Chemometric approaches resulted in a rapid discovery of sensors that can synergistically discriminate up to 12 metal ions with great prediction accuracies. Covalently bound on paper, sensoring scaffolds that were synthesised from the macroarray format can readily be adopted as practical paper-based sensors with great reusability and sensitivity, achieving the limit of detection at low nanomolar level with some repeating spotting. Lastly, the discovered scaffolds were also confirmed to be functional as unbound molecules, thus paving the way for more diverse applications.

## Introduction

The development of efficient sensors for the detection of metal ions is one of the most active research areas in chemical science due to its impact on several disciplines^1–4^. Recent emphasis is on the creation of methods that are amenable for field testing, meaning that rapidness, simplicity and low cost are typically desired^5^. Several stages of a sensor development have been geared towards achieving these properties. For example, microfluidic paper-based analytical devices (μPAD)^6,7^ are a great platform that drastically reduces the cost of detection, thanks to the economical cost of paper. Nevertheless, the selection of readout methods for chemical or biochemical reactions performed on the paper support is also critical to the success of the analysis. Those methods that require sophisticated instrumentation or complicated interpretation are less likely to be widely accepted and in fact contradict to the low-cost nature of the paper-based platform.

Among available methods, fluorescence detection remains an attractive means of measurements, mainly due to its high sensitivity^8,9^. To enable the use of this detection method, reporting molecules capable of exerting changes in either intensity or emission wavelength are required. In the case of metal ion binding, some known mechanisms are generally exploited for observing fluorescence changes. Examples include photoinduced electron transfer (PET) and intramolecular charge transfer (ICT)^10–13^. Anyway, to effectively enhance the paper-based format, sensoring molecules that can provide naked-eye visualisation are essential – this implies that suitable reporters should allow for excitation by commercial black lights with visible-region emissions.

Aminoquinoline is a class of compounds that can fit well with the above requirement. With additional pendants, this type of molecules can accommodate a metal ion, resulting in a change in fluorescence due to PET or ICT^14–20^. Besides, this change is usually a “turn-on” fluorescence, which can theoretically allow unlimited enhancement, thereby having potential to be very sensitive. Hence, various research groups have developed a variety of quinoline derivatives capable of sensing metal ions^21–32^. These usually revealed Zn^2+^ as the main binding partner although some other metal ions were also discovered^22,30^. Interestingly, these studies did not contain much information about structure-property relationships. Our recent study thus aimed to retrace back to investigate the essential core of binding and found that a mere glycine unit was sufficient to achieve clear turn-on fluorescence sensing with Zn^2+^ ion^33^.

In the current study, we aimed to synthesise an expansion set of quinoline probes from the aforementioned essential core by “reversing the discovery paradigm^34^” and thus anticipated to discover some new fluorescent probes in a systematic and rapid manner. Notably, while solid-phase synthesis on polymer beads is a well-established field, our current study was made possible by the use of a carefully-planned macroarray synthesis^35^. This type of technique, originated from the peptide-only synthesis (SPOT synthesis)^36,37^, is a synthesis strategy for small molecules performed on cellulose support. With the spatially-addressable nature, macroarray synthesis allows for the rapid synthesis of multiple compounds on one paper sheet without complex deconvolution. In addition, as similar to other solid-phase techniques, macroarray synthesis was relatively rapid due to the fact that unreacted molecules can be easily washed away without any purification steps. With these unique advantages, previous works have showcased various applications^38–41^ that were directly benefitted from this rapid, low-cost, and reliable method.

Importantly, our current work highlights the first example of how macroarray synthesis can be used to both get access to diverse molecular scaffolds and to perform metal ion sensing on-support without extra cleavage or post-synthesis steps. This accelerated the discovery of sensing molecules even further, and resulted in a set of compounds that, with an aid of chemometrics, can complementarily sense various metal ions, a finding of which would otherwise be improbable or tedious to attain with traditional synthesis. Last but not least, we also demonstrated the utilisation of a Zn^2+^-selective scaffold as a ready-to-use paper-based sensor with great sensitivity and reusability. This was all made possible thanks to the permanent binding nature of the compound to the cellulose support.

## Results and Discussion

**Figure 1:**
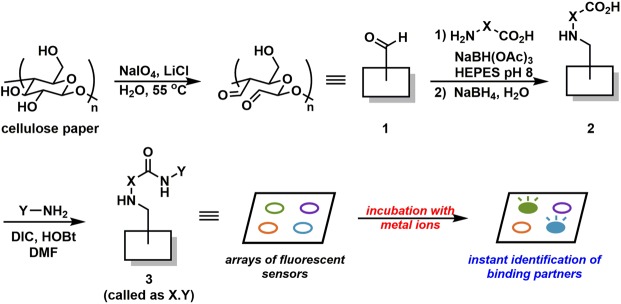
Macroarray synthesis of fluorescent sensors and a subsequent sensing process. DIC = *N*,*N*′-diisopropylcarbodiimide; HOBt = 1-hydroxybenzotriazole; DMF = *N*,*N*′-dimethylformamide; HEPES = *N*-(2-hydroxyethyl)piperazine-*N*′-(2-ethanesulfonic acid).

The synthesis, after some optimisations and/or consideration based on previous other works, can be summarised as follows (Fig. 1). The first step was the treatment of cellulose with NaIO_4_ and LiCl^42,43^. This reaction resulted in the conversion of diol at position 2 and 3 of the glucose repeating unit into the dialdehyde **1**. Thereafter, **1** was subject to reductive amination with a variety of amino acids (**reagent X**, see Supplementary Fig. S1), followed by NaBH_4_ treatment to reduce unreacted surface-bound aldehydes to alcohols. This library **X** consists of readily-available amino acids along with some variations to also probe other effects such as chirality or steric effect due to extended carbon skeletons. The resulting modified surfaces **2** with free carboxyl groups then underwent carbodiimide-mediated couplings with various aminoquinolines and an aminonaphthalimide (**reagent Y**, Supplementary Fig. S1). After washing unbound materials off, **3** can be directly incubated with a solution of a metal ion of interest. Fluorescence response of each surface-bound compound with a metal ion can be easily probed by subjecting the paper sheet with direct illumination from a handheld UV lamp or a transilluminator, thereby revealing active compounds by comparing fluorescence intensity and colour hue with the controls. Also, while the exemplary synthesis in Scheme 1 utilised the spotting method for synthetic steps with the whole-sheet immersion in the sensing step, this overall process can be swapped. This may be more suitable and efficient in certain cases, *e*.*g*., the screening for metal ion partners (by spotting) within a single sheet containing only one sensing scaffold of interest. An example of the exact protocol for the sensing step can be found in the method section. Notably, all synthesis steps could be performed at ambient condition, and the finished cellulose sheets could be kept under a desiccator at ambient temperature for up to at least 2 weeks without deterioration in sensing performance (Supplementary Fig. S2). This reflected a fairly high versatile and durable nature of the whole methodology.

**Figure 2:**
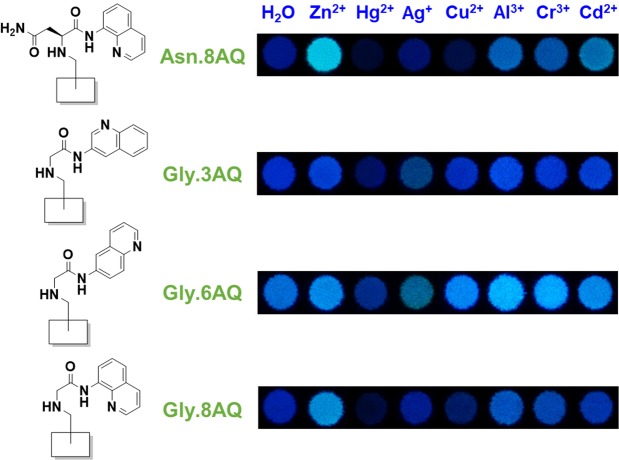
Structures and sensing profiles of four selected fluorophores discovered from the macroarray synthesis against a variety of metal ions.

In this proof-of-concept experiment, we commenced with the screening of the **X** counterpart while keeping the **Y** component to be **8AQ** to allow some comparison with our previous study^33^. The evaluation was performed by observing any fluorescence change when incubated with metal ions including Li^+^, Na^+^, K^+^, Mg^2+^, Ca^2+^, Ba^2+^, Al^3+^, Cr^3+^, Fe^2+^, Co^2+^, Ni^2+^, Cu^2+^, Zn^2+^, Ag^+^, Cd^2+^, Pb^2+^, and Hg^2+^. After obtaining a preliminary result (Supplementary Fig. S3), the **X** components **Gly** and **Asn** were selected for the **Y**-component screening. After compiling obtained results, a set of interesting fluorescent compounds was revealed (Fig. 2). Interestingly, these scaffolds, generally weak or non-fluorescent in water, showed obvious increase in fluorescence upon binding to certain metal ions such as Zn^2+^, Al^3+^, and Cr^3+^, while some metal ions, *e*.*g*., Hg^2+^, can further decrease the fluorescence intensity of certain fluorophores, likely due to the inherent quenching attributes of Hg^2+^ such as enhanced spin-orbit coupling^44,45^. More importantly, the responses of these scaffolds were dependent on specific chemical features present in each scaffold. For example, while the modified surface **Gly**.**8AQ** exhibited strong fluorescence upon binding with Zn^2+^ (believed to be due to a combination of reasons such as the prevention of non-radiative relaxations, *e*.*g*., photoinduced electron transfer from the nitrogen of the -CH_2_-NH- moiety to the quinoline ring, and the restriction of bond rotation), trivalent Al^3+^ and Cr^3+^ seemed to give better fluorescence increase with **Gly**.**3AQ** and **Gly**.**6AQ**, whose aminoquinoline scaffolds are merely regioisomers to that of **Gly**.**8AQ**. **Asn**.**8AQ**, on the other hand, showed strong preference towards both Zn^2+^ and Cd^2+^, the latter of which was never discovered as a metal ion capable of turning on fluorescence of related compounds before. In essence, the ability to obtain such useful information in a relatively short amount of time clearly underscores the fact that macroarray synthesis is indeed an efficient method in revealing new kinds of fluorescent sensors.

**Figure 3:**
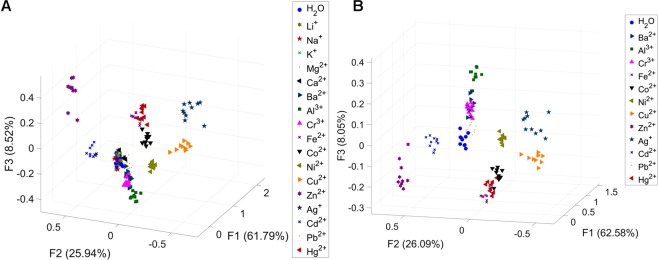
Three-dimensional LDA score plots of the combination of four fluorophores (**Asn**.**8AQ**, **Gly**.**3AQ**, **Gly**.**6AQ**, **Gly**.**8AQ**) in discriminating (**A**) 17 analytes and one blank, and (**B**) 12 analytes with one blank. Ten replicates were performed for each analyte.

To capitalise on the observed signals, chemometric approaches were used to glean more information. First, all fluorescence signals were captured into a digital camera, which were then processed by the imaging software ImageJ. The percentage of red, green, and blue intensities were calculated, and the resulting data were used to create a multidimensional data set with 180 samples (17 metal ions and a blank ×10 replicates) and 12 variances (3 sets of %RGB ×4 fluorophores). Thereafter, the data were analysed by Linear Discriminant Analysis (LDA) and the resulting score plots for all metal ions are shown in Fig. 3, with each group representing the sensing capability of the combination of four sensors (**Asn**.**8AQ**, **Gly**.**3AQ**, **Gly**.**6AQ**, **Gly**.**8AQ**) on each analyte. The LDA score plot showed that most tested ions including Ba^2+^, Al^3+^, Cr^3+^, Fe^2+^, Co^2+^, Ni^2+^, Cu^2+^, Zn^2+^, Ag^+^, Cd^2+^, Pb^2+^, Hg^2+^ can be clearly discriminated by this combined sensing system. Only a handful of group IA and IIA metal ions (Li^+^, Na^+^, K^+^, Mg^2+^, Ca^2+^) could not be distinguished in this system – this is likely due to the well-documented properties of these metal ions such as the harder acid characters and the lack of π-bonding formation capabilities. In addition, LDA with the Jackknife (Leave-One-Out) approach was performed to investigate the predictive ability of the sensing array on the ions. The Jackknife classification yielded 72.35% accuracy in placing all ions into the sensing system (Supplementary Table S1), while the successful prediction was raised up to 93.64% with the exclusion of the aforementioned group IA and IIA metal ions (Supplementary Table S2). This is in good agreement with the LDA score plots.

**Figure 4:**
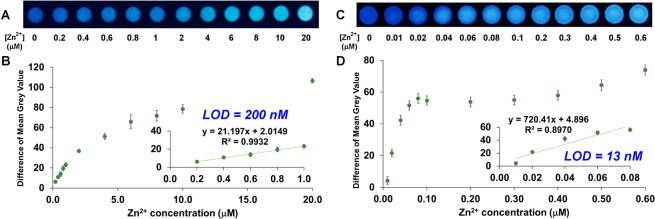
Representative images, along with scattered plots, of fluorescence changes of **Gly**.**8AQ** when incubated with different concentrations of Zn^2+^ ion via one spotting (**A**,**B**) and 10-time repeated spotting (**C**,**D**) of each solution. Calibration plots are also shown as insets in each graph.

Apart from the multiple detections of metal ions by chemometric approaches, a ready-to-use, paper-based device for detecting a single metal ion with naked-eye observation was also fabricated and evaluated for its sensitivity. Specifically, **Gly**.**8AQ** was *in-situ* synthesised on paper support as usual, followed by wax printing to generate confined channels for sample dropping. Thereafter, several concentrations of Zn^2+^ ion were dropped to each channel containing covalently bound **Gly**.**8AQ**, followed by a data collection process (Fig. 4A,B, numerical data in Supplementary Table S3). The digitised data were then used to create a calibration curve, which then revealed that **Gly**.**8AQ** gave a good linear response to the Zn^2+^ concentrations between 0–1 μM. At higher concentrations, the responses reached a plateau. Interestingly, the limit of detection (LOD), obtained from the calibration curve, was found to be about 200 nM (13 ppb), which is comparable to several works using more complicated methods in determining Zn^2+^ ion^46,47^. In addition, the maximum concentration tested, which gave very clear change by naked eyes, was still well below the minimum tolerable limit in bottled waters based on the Code of Federal Regulations at 5 ppm^48^.

This cellulose-bound sensor also offers two additional benefits for sensing application. First, repeating steps of spotting and drying the analyte solution on the detection zone should increase the amount of the analyte in contact with the sensing unit without washing it away. This was found to be a practical way to boost the sensitivity of the sensor by least 10 times (LOD = 13 nM, Fig. 4C,D) when the photographic image was analysed after 10 cycles of spotting and drying steps. The proof of principle here should also be applicable to improve sensitivity of other cellulose-bound sensors or any dryable surface-bound sensors. Second, a reusability test was conducted to exploit the surface-bound nature of the sensor. By simply incubating the Zn^2+^-bound sensor with EDTA, a chelator for Zn^2+^, followed by aqueous washing, the sensor was free and ready to sense Zn^2+^ again. Using this washing process, the cellulose-bound **Gly**.**8AQ** can be easily reused at least 10 times (Supplementary Fig. S4). In addition, the same concept was applied to two other prominent examples including **Asn**.**8AQ** with Zn^2+^ and Cd^2+^ (Supplementary Fig. S5), where the determination of LOD could be done in a similar manner. Taken together, this level of performance is considered to be superior to previous paper-based sensors for Zn^2+^ (Table 1). Clearly, all of them showed higher LOD values/working concentrations while only few studies reported the reusability of their sensors. Hence, the whole process very well demonstrated the utility of the macroarray synthesis in expediting the discovery of compounds with great practicality in sensing applications.

**Table 1:** Comparison of paper-based, fluorescent sensors for Zn^2+^ ion.

Reference	Working concentration (μM)	LOD (μM)	Reusability
^49^	5,000	N. R.	N. R.
^50^	5	N. R.	EDTA wash demonstrated
^51^	1	N. R.	N. R.
^52^	N/A	20	N. R.
^53^	1,000	N. R.	N. R.
^54^	200	N. R.	EDTA wash demonstrated
This work	0.01–1.0	0.01 for 10× repeated spotting	EDTA wash, at least 10 cycles

Working concentration is the concentration of Zn^2+^ ion used for demonstration in each study. This value is used for comparison due to the absence of LOD values in these studies. N/A = not applicable; N. R. = not reported.

**Figure 5:**
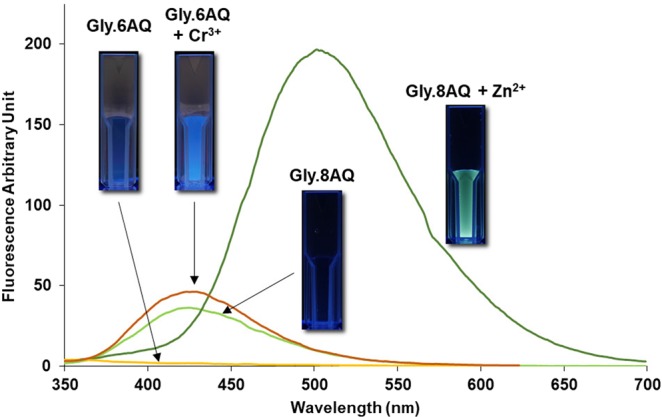
Fluorescence spectra of surface-free **Gly**.**8AQ** (10 μM with/without 50-μM Zn^2+^ – excited at 300 nm) and **Gly**.**6AQ** (10 μM with/without 50-μM Cr^3+^ – excited at 365 nm). Inset: the images of the cuvettes containing the same solutions (but with 100-μM fluorophores with/without 500-μM metal ions) under the illumination of 365-nm light. Note: the photos of **Gly**.**6AQ** solutions were taken with 1-s longer exposure time than that of **Gly**.**8AQ** due to lower fluorescence intensities.

To further confirm the success of the synthesis, and to indirectly characterise the immobilised molecules on support, we synthesised selected compounds containing the suggested scaffolds from the results in Fig. 2 (see the method section)^33^, fully characterised them by NMR spectroscopy and MS, and studied their fluorescence and UV-vis properties. For instance, 2-amino-*N*-(quinolin-8-yl)acetamide (surface-free version of **Gly**.**8AQ**) did show preferential increase in fluorescence upon binding to Zn^2+^ (Fig. 5). The fluorescence titration (Supplementary Fig. S6) also confirmed the gradual decrease of the fluorescence at the emission maximum around 420 nm with concomitant increase in the fluorescence intensity at 500 nm under the higher concentrations of Zn^2+^. UV-vis titration (Supplementary Fig. S7), on the other hand, showed that the absorption maximum around 300 nm was decreased in absorbance with a slight increase in the region around 330–400 nm when the concentrations of Zn^2+^ was increased. On the contrary, it was shown that the fluorescence of 2-amino-*N*-(quinolin-6-yl)acetamide (surface-free version of **Gly**.**6AQ**) was increased upon incubation with Cr^3+^. A similar fluorescence titration (Supplementary Fig. S8) revealed that there was a single emission maximum (450 nm) in this case, whose fluorescence intensity was increased in a direct relationship with the increasing Cr^3+^ concentrations. UV-vis titration (Supplementary Fig. S9) of this sensor-metal ion pair appeared to be slightly complicated, with a general trend being that the absorption around 270–320 nm was significantly increased with higher Cr^3+^ concentrations. This behaviour was also found to be similar for the interaction between the same ligand and Al^3+^ ion (Supplementary Figs S10 and S11). Interestingly, although surface-free **Gly**.**8AQ** could give much higher fluorescence intensity when bound to Zn^2+^ than did all other cases, the turn-on ratio (the fluorescence ratio of Cr^3+^-**Gly**.**6AQ** complex over free **Gly**.**6AQ**) was slightly higher than that of **Gly**.**8AQ** with Zn^2+^ (ca. 25 folds vs 22 folds) due to much lower background in free **Gly**.**6AQ**. This indicated that both can function well as sensors for their respective metal ion partners, and suggested that the synthetic strategy used in this study was an effective one to rapidly evaluate fluorescence properties of a variety of molecular scaffolds.

Last but not least, titration experiments via MS and NMR were also conducted to further confirm the binding interactions between selected pairs of ligands and metal ions. For MS titrations, as shown in Supplementary Fig. S12, surface-free **Gly**.**8AQ** exhibited both 1:1 and 2:1 binding modes (ligand:metal ion) when Zn^2+^ ion was sub-stoichiometric amounts, and 1:1 binding seemed to be dominant when the molar ratio was around 1:1. On the other hand, **Gly**.**6AQ** interacted with Cr^3+^ in a more complicated manner, showing only 3:2 binding mode (ligand:metal ion) once the molar ratio reached 1:1 (Supplementary Fig. S13). For NMR titration, the set of **Gly**.**8AQ** and Zn^2+^ showed several apparent shifts, with the most notable one being the methylene protons (starting at around 3.5 ppm) when more metal ion was added into the solution (Supplementary Fig. S14). All of these evidences clearly pointed out that there were indeed some significant bindings between the ligands and the metal ions used in this work.

In conclusion, the macroarray synthesis approach was implemented herein to rapidly generate a variety of fluorophores, which were readily amenable to direct screenings to discover metal ion sensors. Using chemometric approaches, combined fluorophore systems could predict several metal ions with high prediction accuracies, while the fabrication of a single fluorophore to detect Zn^2+^ gave a practical paper-based sensor with great sensitivity and reusability. Lastly, this information could be reversed to yield typical solution-based sensors, hence demonstrating that the synthesis methodology is also a very useful tool for rapid discovery of even larger and more diverse libraries of fluorescent sensors.

## Methods

### General information

All reagents were purchased from commercial sources and used without further purification. All metal ions used in this study were in the form of nitrate salts except Fe^2+^, where the acetate salt was used instead. Paper used in this study was Whatman™ Grade 1 Qualitative Filter Paper, which was used without further treatment. Solutions were made with Milli-Q water from ultrapure water systems with a Millipak 40 filter unit (0.22 μm, Millipore, USA). NMR spectra were acquired on a Bruker NMR spectrometer at 400 MHz (^1^H) and 100 MHz (^13^C). The high resolution mass spectra were obtained from an electrospray ionisation MS (microTOF, Bruker Datlonics). Absorption spectra were measured by using Varian Cary 50 UV-vis spectrophotometer. Fluorescence spectra were recorded on a Varian Cary Eclipse spectrofluorometer. Mass titration spectra were obtained from an electrospray ionisation MS (Thermo Scientific TSQ Quantum EMR triple quadrupole MS) with the following parameters: positive mode; spray voltage at 3000 V; capillary temperature at 300 °C; tube lens offset at 84 V).

### Generation of aldehyde functional groups on paper

Paper sheets were washed with a series of solvents (DMF, 10 min, 3x, then MeOH, 10 min, 3x) prior to chemical functionalization. Thereafter, paper sheets were immersed into a solution of NaIO_4_ (10 mM) and LiCl (100 mM) premixed in 10 mL H_2_O at 55 °C for 1 h. This was followed by washing with Milli-Q water (10 min, 3x), MeOH (10 min, 3x), and air-dried.

### Reductive amination with reagent X

A solution of each reagent X (amino acids or dipeptides, 1 mmol) with NaBH(OAc)_3_ (212 mg, 1 mmol) was prepared by adding 1 mL of 10-mM HEPES pH 8 solution into pre-weighed reagents, resulting in a 1 M solution. In the case of incompletely soluble reagents (judged by observing precipitate after 20-min sonication), only supernatant was withdrawn and used. The resulting solution was spotted (2 μL) onto desired areas on aldehyde-terminated papers (surface **1** in the main article). The sheet was incubated at room temperature for 10 min, and the spotting was repeated two more times. After washing (Milli-Q water (10 min, 3x), then MeOH (10 min, 3x)), the paper sheets were immersed into a solution of NaBH_4_ (100 mM) for 20 min at room temperature. This again was followed by a series of washing (Milli-Q water (10 min, 3x), then MeOH (10 min, 3x)).

### Carbodiimide coupling with reagent Y

A solution of each reagent Y (aminoquinolines or an aminonaphthalimide, 1 mmol) with HOBt (154 mg, 1 mmol) was prepared by adding 1 mL of DMF into pre-weighed reagents. The resulting solution was mixed with DIC (126 μL, 1 mmol) and the new mixture was immediately used for spotting (2 μL) onto desired areas on carboxyl-terminated papers (surface **2** in the main article). The sheet was incubated at room temperature for 5 min, and the spotting was repeated two more times. The process ended by washing the sheets with HEPES buffer pH 5 solution (10 min, 1x), HEPES buffer pH 7 solution (10 min, 1x), DMF (10 min, 5x), and then MeOH (10 min, 3x). After air drying, the paper sheets were ready for metal ion sensing.

### Final finishing with wax printing

If desired, finished sensors can be patterned with hydrophobic wax as shown in our studies. This was done by a Xerox ColorQube 8870 wax printer to create circular patterns (diameter at 7 mm) on paper (which was designed by Adobe Photoshop). Heating at 110 °C for 30 sec on a hotplate was then performed to melt the wax and create the final hydrophobic patterns (diameter of around 5 mm).

### Probing fluorescence response with various metal ions

#### First option – immersion method

Each finished paper sheet was immersed in a solution of a metal ion (10 μM in HEPES buffer pH 7). The solution was incubated for 30 min. The sheet was air-dried and exposed to UV light. A handheld UV lamp was used to illuminate directly on the paper for fluorescence observation.

#### Second option – spotting method

Each desired area on a finished paper sheet was spotted (2 µL) with a solution of a metal ion (10 μM in HEPES buffer pH 7). The sheet was incubated for 10 min. The sheet was air-dried and exposed to UV light. A handheld UV lamp was used to illuminate directly on the paper for fluorescence observation.

### Digital conversion and quantitative analysis of fluorescence signals

Fluorescence signals were captured by using a digital camera (model Panasonic DMC-GF7) with the following camera setting: ISO-200, f/5.0, and 2 s exposure time. The illuminator (TCP-20.LM, Vilber Lourmat, Germany) provided the source of 365-nm light. Similar to other reaction steps, photos were taken under ambient condition, with a handmade cardboard box with a hole to fit the front lens of the camera. These images were then extracted for their RGB values and analysed with ImageJ (http://imagej.nih.gov/ij/). Since the areas to be analysed were usually circular, we employed the circular region of interest (ROI) to measure the signal intensity. Note that the area was smaller than the average size of each signal area (by 78%) to ensure that all measured area contained some signal intensity. The integrated density as calculated by ImageJ can then be obtained. For chemometric experiments, all raw R, G, and B values were used, while the LOD experiment instead made use of mean grey values and their difference values.

### Chemometrics

ImageJ software was used to extract all red, green, and blue color intensity from each reaction data points. These RGB values were then used to calculate for percentage of each color by using the formula: %R = (R/(R + G + B) × 100. The same can be done for %G and %B.

Linear discriminant analysis (LDA) was used with a data set consisting of 180 samples (17 metal ions and blank ×10 replicates) and 12 variances (3 sets of %RGB ×4 fluorophores). The calculation and plotting were performed using in-house codes based on MATLAB R2018a. Linear discriminant analysis (LDA) for the Jackknife classification provided percent classification accuracies. This is represented in the tables below (Tables S1 and S2).

### Synthesis of representative fluorophores

Synthesis of (2-amino-*N*-(quinolin-8-yl)acetamide) (unbound **Gly**.**8AQ**). A mixture of 8-aminoquinoline (500 mg, 3.47 mmol), DMAP (21.18 mg, 0.17 mmol) and Boc-Gly-OH (1.22 g, 6.94 mmol) in dichloromethane (30 mL) was stirred in ice bath (~0 °C) for 30 minutes, followed by an addition of EDC·HCl (1.33 g, 6.94 mmol). The reaction mixture was stirred at 0 °C for 2 hours and stirred overnight at room temperature. The crude was extracted with NH_4_Cl (aq). The organic layer was dried over MgSO_4_, filtered and concentrated under vacuum. The crude product was purified on a column chromatography using ethyl acetate: hexane (2:3) as an eluent to afford (2-Boc-amino-*N*-(quinolin-8-yl)acetamide) as a white solid (910 mg, 3 mmol, 87%). This compound was stirred overnight with trifluoroacetic acid (0.46 mL, 6 mmol) in dichloromethane 20 mL at room temperature. The crude was extracted with saturated NaHCO_3_. The organic layer was dried over MgSO_4_, filtered and concentrated under vacuum. The crude product was purified on a column chromatography using ethyl acetate as an eluent to afford (2-amino-*N*-(quinolin-8-yl)acetamide) as a yellow solid (free base form, 437 mg, 2.2 mmol, 72%). ^1^H NMR (400 MHz, DMSO) δ 11.62 (s, 1H), 8.92 (dd, *J* = 4.0, 1.1 Hz, 1H), 8.75 (d, *J* = 7.5 Hz, 1H), 8.39 (d, *J* = 8.2 Hz, 1H), 7.67–7.54 (m, 3H), 3.41 (s, 2H), 2.40 (s, 2H). ^13^C NMR (101 MHz, DMSO) δ 172.2, 148.9, 138.1, 136.4, 134.2, 127.8, 127.0, 122.0, 121.5, 115.3, 45.6, 39.5. MS (ESI); m/z calculated for [C_11_H_11_N_3_O + Na]^+^ is 224.07943; found 224.07959 [M + Na]^+^. The spectra images of ^1^H and ^13^C NMR can also be found in Supplementary Fig. S15.

Synthesis of (2-amino-*N*-(quinolin-6-yl)acetamide) (unbound, TFA salt of **Gly**.**6AQ**). A mixture of 6-aminoquinoline (500 mg, 3.47 mmol), DMAP (21.18 mg, 0.17 mmol) and Boc-Gly-OH (1.22 g, 6.94 mmol) in dichloromethane (30 mL) was stirred in ice bath (~0 °C) for 30 minutes, followed by an addition of EDC·HCl (1.33 g, 6.94 mmol). The reaction mixture was stirred at 0 °C for 2 hours and stirred overnight at room temperature. The crude was extracted with NH_4_Cl (aq). The organic layer was dried over MgSO_4_, filtered and concentrated under vacuum. The crude product was purified on a column chromatography using ethyl acetate as an eluent to afford (2-Boc-amino-*N*-(quinolin-6-yl)acetamide) as a white solid (700 mg, 2.32 mmol, 67%). Thereafter, this compound was stirred overnight with trifluoroacetic acid (0.36 mL, 4.64 mmol) in dichloromethane 20 mL at room temperature. The crude product was left under vacuum for about 2 hours at 60 °C to afford 2-oxo-2-(quinolin-6-ylamino)ethan-1-aminium 2,2,2-trifluoroacetate as a yellow solid (400 mg, 1.27 mmol, 55%). ^1^H NMR (400 MHz, DMSO) δ 11.20 (s, 1H), 9.09 (d, *J* = 4.7 Hz, 1H), 8.92 (d, *J* = 8.2 Hz, 1H), 8.58 (d, *J* = 1.2 Hz, 1H), 8.35 (s, 3H), 8.22 (d, *J* = 9.2 Hz, 1H), 8.05 (dd, *J* = 9.2, 1.6 Hz, 1H), 7.89 (dd, *J* = 7.5, 5.6 Hz, 1H). ^13^C NMR (101 MHz, DMSO) δ 165.9, 159.0 (q, *J* = 36.5 Hz, TFA), 145.3, 143.0, 138.2, 137.5, 129.3, 126.6, 124.4, 122.4, 116.1 (q, *J* = 292.2 Hz, TFA), 115.4, 41.4. MS (ESI); m/z calculated for [C_11_H_11_N_3_O]^+^ is 202.09749; found 202.09820 [M + H]^+^. The spectra images of ^1^H and ^13^C NMR can also be found in Supplementary Fig. S16.

The synthesis of (2-amino-*N*-(quinolin-3-yl)acetamide) (unbound, TFA salt of **Gly**.**3AQ**) and 2-amino-*N*^1^-(quinolin-8-yl)succinamide) (unbound, TFA salt of **Asn**.**8AQ**) were done in a similar manner, and their NMR spectra were shown in Supplementary Figs S17 and S18, respectively.

## Supplementary information

Supplementary information

## Data Availability

All data that are related to this study but not presented herein are available from the corresponding author upon request.

## References

[1] El-Kady AA, Abdel-Wahhab MA (2018). Occurrence of trace metals in foodstuffs and their health impact. Trends Food Sci. Technol..

[2] Khalid Sana, Shahid Muhammad, Natasha, Bibi Irshad, Sarwar Tania, Shah Ali, Niazi Nabeel (2018). A Review of Environmental Contamination and Health Risk Assessment of Wastewater Use for Crop Irrigation with a Focus on Low and High-Income Countries. International Journal of Environmental Research and Public Health.

[3] Planchart A, Green A, Hoyo C, Mattingly CJ (2018). Heavy Metal Exposure and Metabolic Syndrome: Evidence from Human and Model System Studies. Curr. Environ. Health Rep..

[4] Vaananen K, Leppanen MT, Chen XP, Akkanen J (2018). Metal bioavailability in ecological risk assessment of freshwater ecosystems: From science to environmental management. Ecotoxicol. Environ. Saf..

[5] Rull-Barrull J, d’Halluin M, Le Grognec E, Felpin F-X (2016). Chemically-modified cellulose paper as smart sensor device for colorimetric and optical detection of hydrogen sulfate in water. Chem. Commun..

[6] Martinez AW, Phillips ST, Butte MJ, Whitesides GM (2007). Patterned paper as a platform for inexpensive, low-volume, portable bioassays. Angew. Chem. Int. Ed..

[7] Yetisen AK, Akram MS, Lowe CR (2013). Paper-based microfluidic point-of-care diagnostic devices. Lab Chip.

[8] Carter KP, Young AM, Palmer AE (2014). Fluorescent Sensors for Measuring Metal Ions in Living Systems. Chem. Rev..

[9] Li, M., Li, X., Xiao, H.-N. & James, T. D. Fluorescence Sensing with Cellulose-Based Materials. *Chemistry Open*, 685–696 (2017). 10.1002/open.201700133 PMC5715359 29226055

[10] Valeur B, Leray I (2000). Design principles of fluorescent molecular sensors for cation recognition. Coord. Chem. Rev..

[11] Xu Z, Xiao Y, Qian X, Cui J, Cui D (2005). Ratiometric and Selective Fluorescent Sensor for CuII Based on Internal Charge Transfer (ICT). Org. Lett..

[12] Wang J, Qian X (2006). A Series of Polyamide Receptor Based PET Fluorescent Sensor Molecules:  Positively Cooperative Hg^2+^ Ion Binding with High Sensitivity. Org. Lett..

[13] Chang K-C, Su I-H, Senthilvelan A, Chung W-S (2007). Triazole-Modified Calix[4]crown as a Novel Fluorescent On–Off Switchable Chemosensor. Org. Lett..

[14] Panda D, Datta A (2006). The role of the ring nitrogen and the amino group in the solvent dependence of the excited-state dynamics of 3-aminoquinoline. J. Chem. Phys..

[15] Xue L, Liu C, Jiang H (2009). Highly Sensitive and Selective Fluorescent Sensor for Distinguishing Cadmium from Zinc Ions in Aqueous Media. Org. Lett..

[16] Cao XW, Lin WY, He LW (2011). A Near-Infrared Fluorescence Turn-On Sensor for Sulfide Anions. Org. Lett..

[17] Liu ZP, Zhang CL, Wang XQ, He WJ, Guo ZJ (2012). Design and Synthesis of a Ratiometric Fluorescent Chemosensor for Cu(II) with a Fluorophore Hybridization Approach. Org. Lett..

[18] Ma Y (2014). A highly sensitive and selective ratiometric fluorescent sensor for Zn^2+^ ion based on ICT and FRET. Dyes Pigments.

[19] Vongnam K, Muangnoi C, Rojsitthisak P, Sukwattanasinitt M, Rashatasakhon P (2016). A highly selective turn-on fluorescent sensor for glucosamine from amidoquinoline-napthalimide dyads. Biosens. Bioelectron..

[20] Liu JR (2017). Synthesis and *in vitro* evaluation of new fluorinated quinoline derivatives with high affinity for PDE5: Towards the development of new PET neuroimaging probes. Eur. J. Med. Chem..

[21] Zhang Y, Guo XF, Si WX, Jia LH, Qian XH (2008). Ratiometric and water-soluble fluorescent zinc sensor of carboxamidoquinoline with an alkoxyethylamino chain as receptor. Org. Lett..

[22] Dong Z (2009). Quinoline group grafted carbon nanotube fluorescent sensor for detection of Cu^2+^ ion. Appl. Surf. Sci..

[23] Zhu JF, Yuan H, Chan WH, Lee AWM (2010). A FRET fluorescent chemosensor SPAQ for Zn^2+^ based on a dyad bearing spiropyran and 8-aminoquinoline unit. Tetrahedron Lett..

[24] Pal P (2011). Fluorescence Sensing of Zinc(II) Using Ordered Mesoporous Silica Material (MCM-41) Functionalized with N-(Quinolin-8-yl)-2-[3-(triethoxysilyl)propylamino]acetamide. ACS Appl. Mater. Interfaces.

[25] Zhou XB (2011). Ratiometric fluorescent Zn^2+^ chemosensor constructed by appending a pair of carboxamidoquinoline on 1,2-diaminocyclohexane scaffold. Tetrahedron.

[26] Zhang Y (2012). Substituent-dependent fluorescent sensors for zinc ions based on carboxamidoquinoline. Dalton Trans..

[27] Dong ZP, Guo YP, Tian X, Ma JT (2013). Quinoline group based fluorescent sensor for detecting zinc ions in aqueous media and its logic gate behaviour. J. Lumin..

[28] Goswami S (2013). Ratiometric and absolute water-soluble fluorescent tripodal zinc sensor and its application in killing human lung cancer cells. Analyst.

[29] Lee HG (2013). Zinc selective chemosensors based on the flexible dipicolylamine and quinoline. Inorg. Chim. Acta.

[30] Ma Y, Wang F, Kambam S, Chen XQ (2013). A quinoline-based fluorescent chemosensor for distinguishing cadmium from zinc ions using cysteine as an auxiliary reagent. Sens. Actuators B Chem..

[31] Dong Z, Le X, Zhou P, Dong C, Ma J (2014). An “off-on-off” fluorescent probe for the sequential detection of Zn^2+^ and hydrogen sulfide in aqueous solution. New J. Chem..

[32] Pradhan AB (2015). A highly selective fluorescent sensor for zinc ion based on quinoline platform with potential applications for cell imaging studies. Polyhedron.

[33] Boonkitpatarakul K (2018). An 8-aminoquinoline derivative as a molecular platform for fluorescent sensors for Zn(II) and Cd(II) ions. J. Lumin..

[34] Mello JV, Finney NS (2005). Reversing the discovery paradigm: A new approach to the combinatorial discovery of fluorescent chemosensors. J. Am. Chem. Soc..

[35] Blackwell HE (2006). Hitting the SPOT: small-molecule macroarrays advance combinatorial synthesis. Curr. Opin. Chem. Biol..

[36] Hilpert K, Winkler DFH, Hancock REW (2007). Peptide arrays on cellulose support: SPOT synthesis, a time and cost efficient method for synthesis of large numbers of peptides in a parallel and addressable fashion. Nat. Protoc..

[37] Volkmer R (2009). Synthesis and Application of Peptide Arrays: Quo Vadis SPOT Technology. Chem Bio Chem.

[38] Bowman MD, Jeske RC, Blackwell HE (2004). Microwave-accelerated SPOT-synthesis on cellulose supports. Org. Lett..

[39] Lin Q, O’Neill JC, Blackwell HE (2005). Small molecule macroarray construction via Ugi four-component reactions. Org. Lett..

[40] Bowman MD, Jacobson MM, Blackwell HE (2006). Discovery of Fluorescent Cyanopyridine and Deazalumazine Dyes Using Small Molecule Macroarrays. Org. Lett..

[41] Praneenararat T, Geske GD, Blackwell HE (2009). Efficient Synthesis and Evaluation of Quorum-Sensing Modulators Using Small Molecule Macroarrays. Org. Lett..

[42] Sirvio J, Hyvakko U, Liimatainen H, Niinimaki J, Hormi O (2011). Periodate oxidation of cellulose at elevated temperatures using metal salts as cellulose activators. Carbohydr. Polym..

[43] Noor MO, Krull UJ (2013). Paper-Based Solid-Phase Multiplexed Nucleic Acid Hybridization Assay with Tunable Dynamic Range Using Immobilized Quantum Dots As Donors in Fluorescence Resonance Energy Transfer. Anal. Chem..

[44] Descalzo AB, Martínez-Máñez R, Radeglia R, Rurack K, Soto J (2003). Coupling Selectivity with Sensitivity in an Integrated Chemosensor Framework:  Design of a Hg^2+^ -Responsive Probe, Operating above 500 nm. J. Am. Chem. Soc..

[45] Rurack K (2001). Flipping the light switch ‘ON’ – the design of sensor molecules that show cation-induced fluorescence enhancement with heavy and transition metal ions. Spectrochim. Acta, Pt. A: Mol. Biomol. Spectrosc..

[46] Ressalan S, Iyer CSP (2004). Review of spectrofluorimetric methods for the determination of copper, nickel and zinc. Rev. Anal. Chem..

[47] Pohl P (2008). Determination of metal content in honey by atomic absorption and emission spectrometries. TrAC, Trends Anal. Chem..

[48] eCFR - Code of Federal Regulations; Bottled water, https://www.ecfr.gov/cgi-bin/text-idx?SID=a345042f6bd438d7949b0270596b60fd&mc=true&node=se21.2.165_1110&rgn=div8 (2018).

[49] Udhayakumari D, Saravanamoorthy S, Ashok M, Velmathi S (2011). Simple imine linked colorimetric and fluorescent receptor for sensing Zn^2+^ ions in aqueous medium based on inhibition of ESIPT mechanism. Tetrahedron Lett..

[50] Hu J-H, Sun Y, Qi J, Li Q, Wei T-B (2017). A new unsymmetrical azine derivative based on coumarin group as dual-modal sensor for CN− and fluorescent “OFF–ON” for Zn^2+^. Spectrochim. Acta, Pt. A: Mol. Biomol. Spectrosc..

[51] Diao H, Guo L, Liu W, Feng L (2018). A novel polymer probe for Zn(II) detection with ratiometric fluorescence signal. Spectrochim. Acta, Pt. A: Mol. Biomol. Spectrosc..

[52] Wen X, Wang Q, Fan Z (2018). Highly selective turn-on fluorogenic chemosensor for Zn(II) detection based on aggregation-induced emission. J. Lumin..

[53] Pang B-J, Li C-R, Yang Z-Y (2018). A novel chromone and rhodamine derivative as fluorescent probe for the detection of Zn(II) and Al(III) based on two different mechanisms. Spectrochim. Acta, Pt. A: Mol. Biomol. Spectrosc..

[54] Chae JB (2019). Fluorescent determination of zinc by a quinoline-based chemosensor in aqueous media and zebrafish. Spectrochim. Acta, Pt. A: Mol. Biomol. Spectrosc..

