# Supplementary material for: Rapid Discovery and Structure-Property Relationships of Metal-Ion Fluorescent Sensors via Macroarray Synthesis
Source: Sci Rep. 2019 Jul 17;9:10390. doi: 10.1038/s41598-019-46783-8 (PMC6637192; doi:10.1038/s41598-019-46783-8)
Supplement: Supplementary file 1 — Supplementary information [file 41598_2019_46783_MOESM1_ESM.pdf]

SUPPLEMENTARY INFORMATION

Rapid Discovery and Structure-Property Relationships of Metal-Ion Fluorescent Sensors via Macroarray Synthesis

Apiwat Promchat,[a,b] Kanet Wongravee,[a,c] Mongkol Sukwattanasinitt,[a,b] and Thanit Praneenararat[a,d,]*

[a] Department of Chemistry, Faculty of Science, Chulalongkorn University, Phayathai Rd., Pathumwan, Bangkok, Thailand, 10330.

[b] Nanotec-CU Center of Excellence on Food and Agriculture, Department of Chemistry, Faculty of Science, Chulalongkorn University, Phayathai Rd., Pathumwan, Bangkok, Thailand, 10330.

[c] Sensor Research Unit, Department of Chemistry, Faculty of Science, Chulalongkorn University, Phayathai Rd., Pathumwan, Bangkok, Thailand, 10330.

[d] The Chemical Approaches for Food Applications Research Group, Faculty of Science, Chulalongkorn University, Phayathai Rd., Pathumwan, Bangkok, Thailand, 10330.

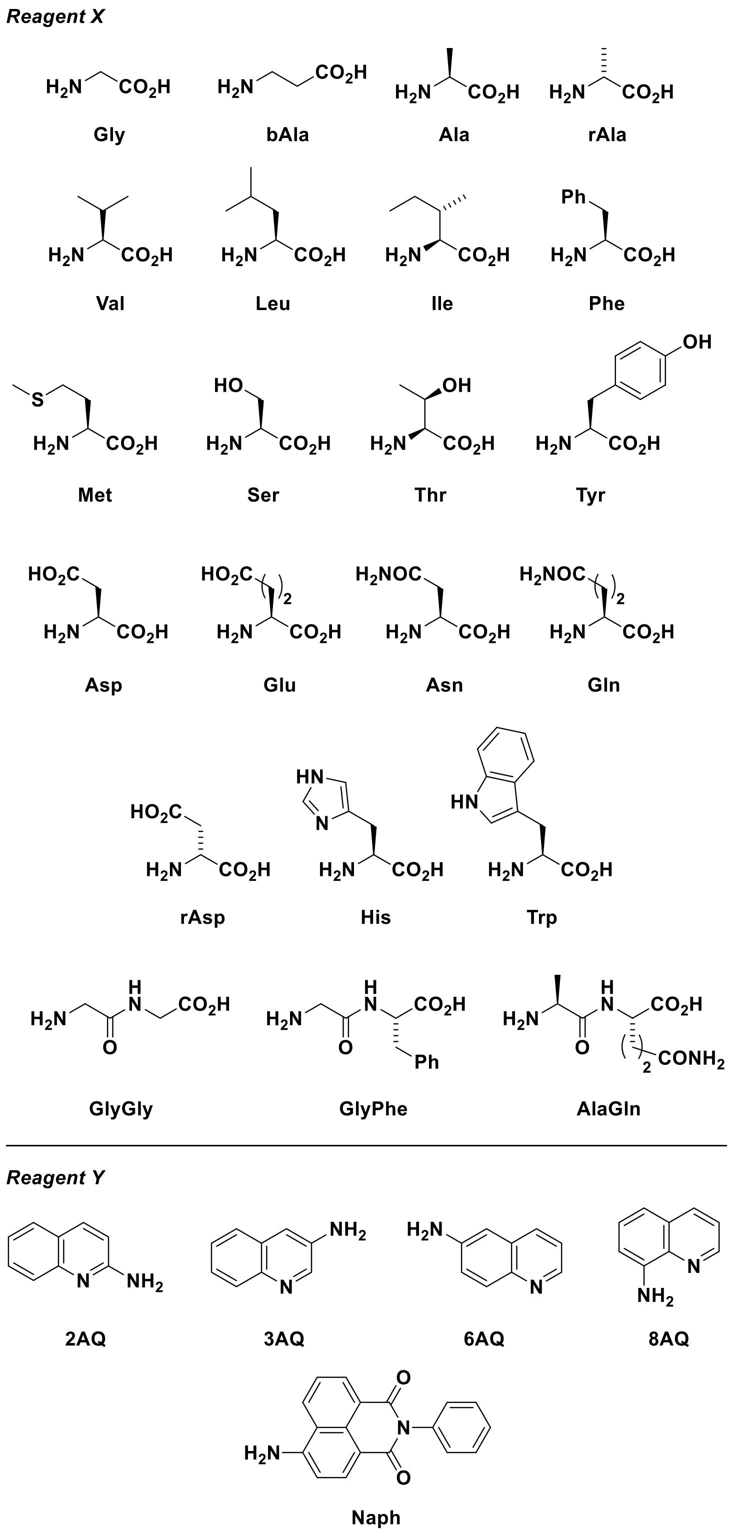

Figure S1. Structures of **Reagent X** and **Reagent Y** used in this study.

Durability testing of the finished paper-based sensors in sensing selected ions

The images under UV-light of all paper-based sensors were converted into numerical values as explained in the experimental methods above. The concentrations of Cr^{3+} and Zn^{2+} were the same as in the selectivity test for LDA (Figure 3).

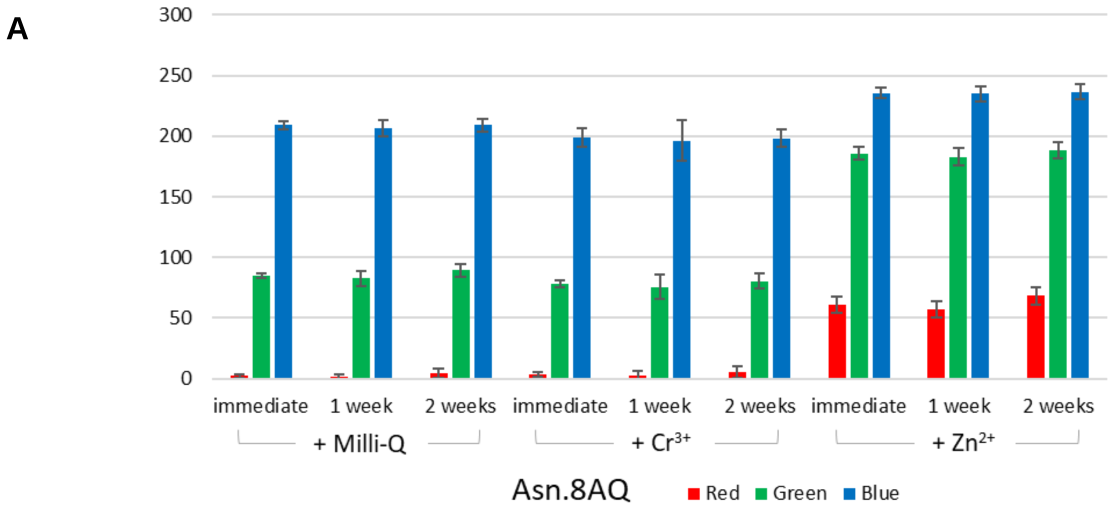

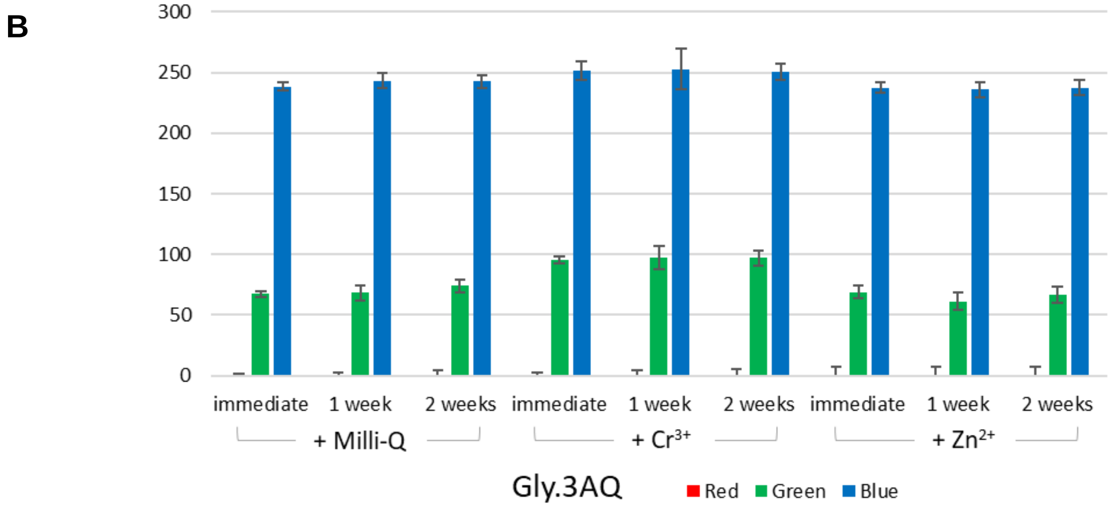

C

D

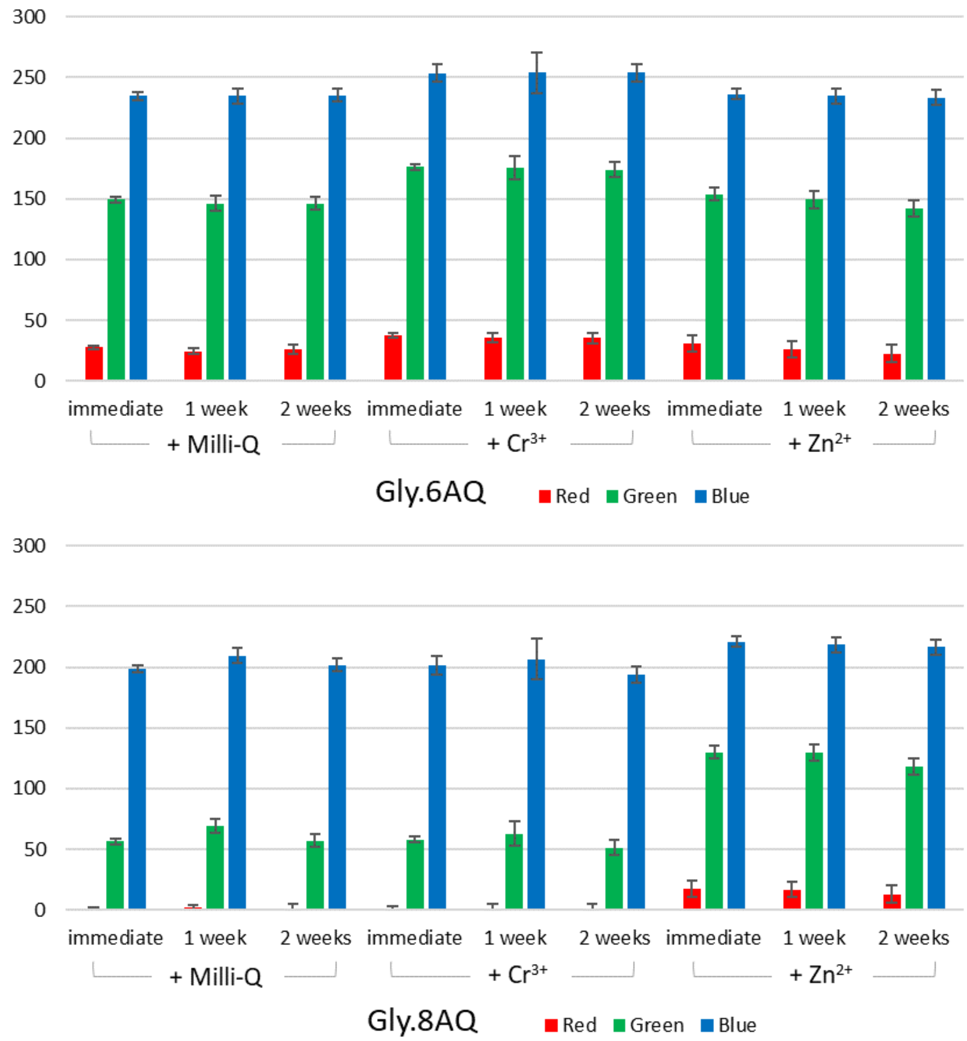

Figure S2. Bar graphs representing intensities of each color channel from A) **Asn.8AQ**, B) **Gly.3AQ**, C) **Gly.6AQ**, D) **Gly.8AQ** in responding to Milli-Q water, Cr^{3+}, and Zn^{2+} over the period of two weeks.

Preliminary screenings of various sensors with a set of metal ions

Gly.8AQ

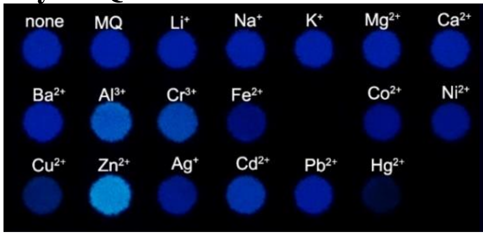

bAla.8AQ

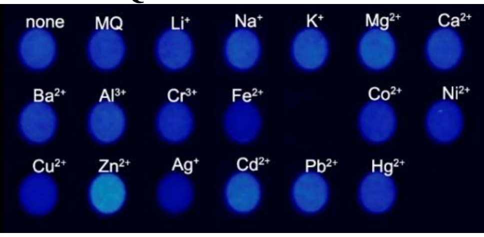

Ala.8AQ

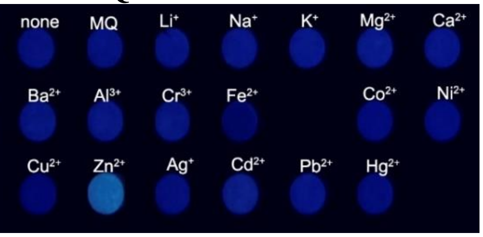

rAla.8AQ

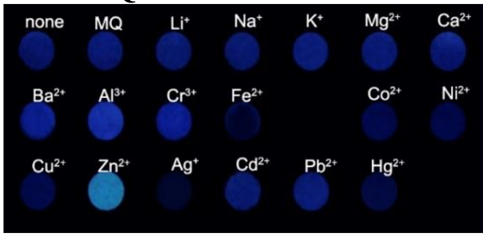

Val.8AQ

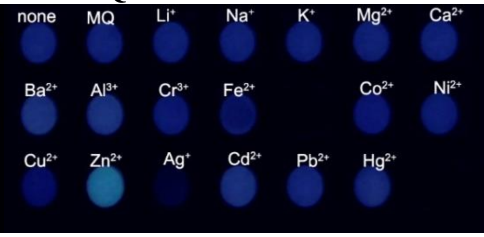

Leu.8AQ

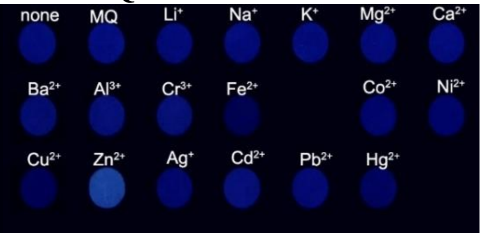

Ile.8AQ

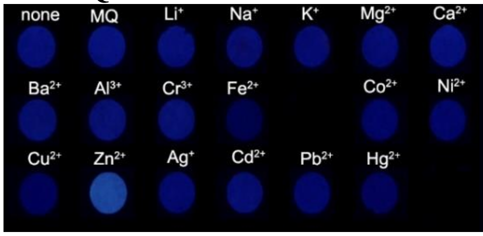

Phe.8AQ

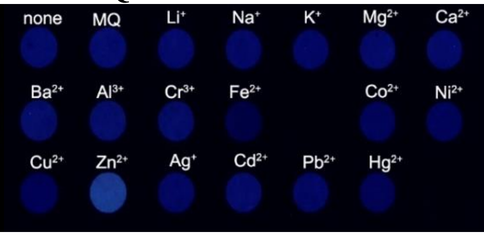

Met.8AQ

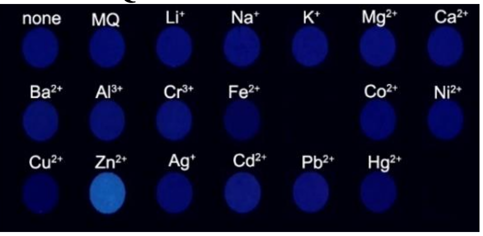

Ser.8AQ

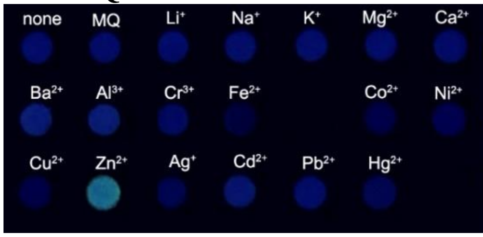

Thr.8AQ

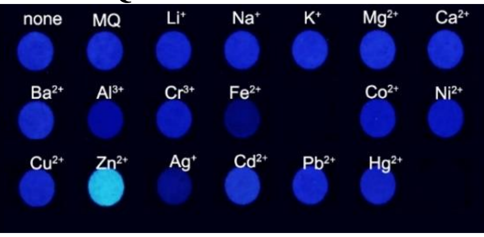

Tyr.8AQ

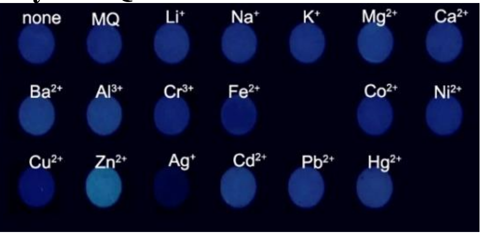

Asp.8AQ

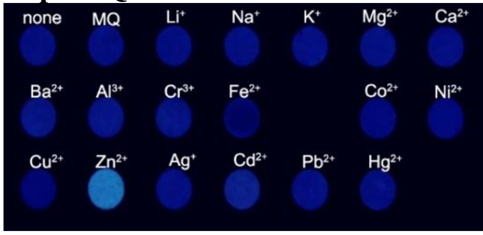

Glu.8AQ

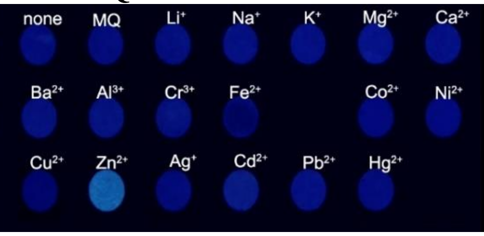

Asn.8AQ

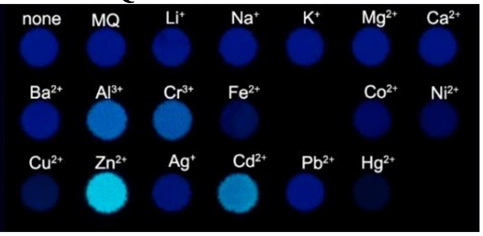

Gln.8AQ

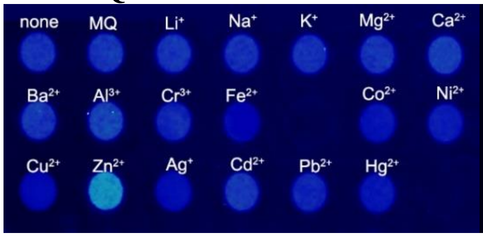

rAsp.8AQ

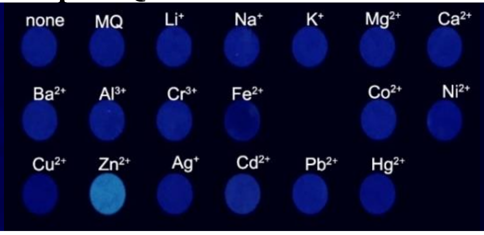

His.8AQ

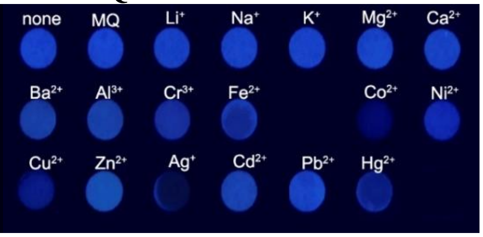

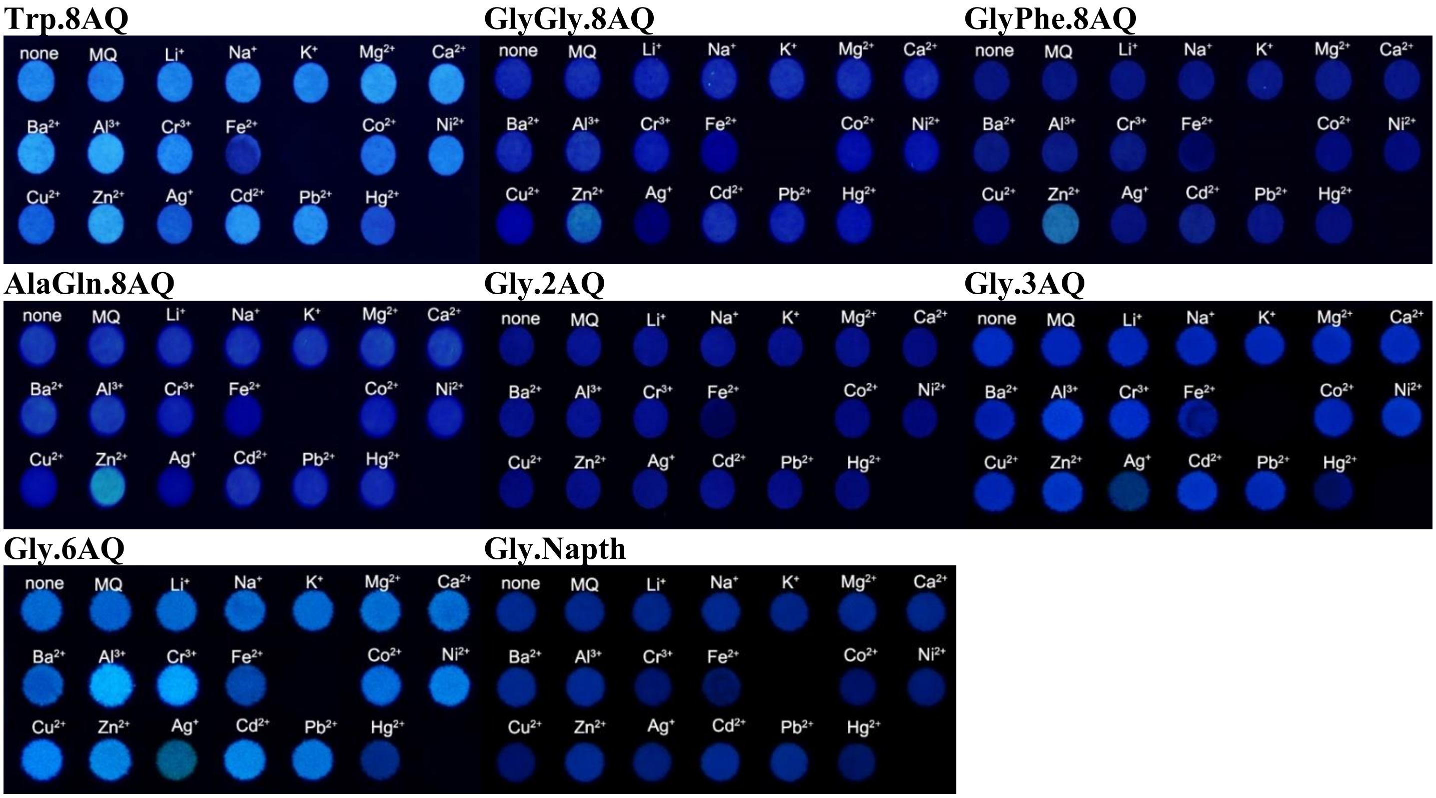

Figure S3. Preliminary fluorescence screening of all scaffolds synthesised via macroarray synthesis against selected metal ions.

Additional chemometric results

Table S1. A Jackknife (leave one out cross-validation) classification table for the ions based on the discrimination performance of the combined sensors (**Asn.8AQ, Gly.3AQ, Gly.6AQ, Gly.8AQ)** in discriminating 17 metal ions and a blank. The prediction accuracy was determined to be 72.35%.

Analytes	H_2O	Li^+	Na^+	K^+	Mg^{2+}	Ca^{2+}	Ba^{2+}	Al^{3+}	Cr^{3+}	Fe^{2+}	Co^{2+}	Ni^{2+}	Cu^{2+}	Zn^{2+}	Ag^+	Cd^{2+}	Pb^{2+}	Hg^{2+}	%Class.
H_2O	0	2	1	4	2	1	0	0	0	0	0	0	0	0	0	0	0	0	0
Li^+	1	2	3	1	2	1	0	0	0	0	0	0	0	0	0	0	0	0	20
Na^+	1	2	4	2	0	1	0	0	0	0	0	0	0	0	0	0	0	0	40
K^+	0	2	1	3	0	4	0	0	0	0	0	0	0	0	0	0	0	0	30
Mg^{2+}	1	3	0	0	3	2	0	0	0	0	0	0	0	0	0	0	1	0	30
Ca^{2+}	0	3	1	2	1	2	0	0	0	0	0	0	0	0	0	0	1	0	20
Ba^{2+}	0	0	0	0	0	0	6	0	2	0	0	0	0	0	0	0	2	0	60
Al^{3+}	0	0	0	0	0	0	1	9	0	0	0	0	0	0	0	0	0	0	90
Cr^{3+}	0	0	0	0	0	0	2	0	8	0	0	0	0	0	0	0	0	0	80
Fe^{2+}	0	0	0	0	0	0	0	0	0	9	0	0	0	0	0	0	0	1	90
Co^{2+}	0	0	0	0	0	0	0	0	0	0	10	0	0	0	0	0	0	0	100
Ni^{2+}	0	0	0	0	0	0	0	0	0	0	0	10	0	0	0	0	0	0	100
Cu^{2+}	0	0	0	0	0	0	0	0	0	0	0	0	10	0	0	0	0	0	100
Zn^{2+}	0	0	0	0	0	0	0	0	0	0	0	0	0	10	0	0	0	0	100
Ag^+	0	0	0	0	0	0	0	0	0	0	0	0	0	0	10	0	0	0	100
Cd^{2+}	0	0	0	0	0	0	0	0	0	0	0	0	0	0	0	10	0	0	100
Pb^{2+}	0	0	0	0	1	1	0	0	0	0	0	0	0	0	0	0	8	0	80
Hg^{2+}	0	0	0	0	0	0	0	0	0	1	0	0	0	0	0	0	0	9	90

(%class. = %classifications)

Table S2. A Jackknife (leave one out cross-validation) classification table for the ions based on the discrimination performance of the combined sensors (**Asn.8AQ, Gly.3AQ, Gly.6AQ, Gly.8AQ)** in discriminating 12 metal ions and a blank. The prediction accuracy was determined to be 93.64%.

Analytes	H_2O	Ba^{2+}	Al^{3+}	Cr^{3+}	Fe^{2+}	Co^{2+}	Ni^{2+}	Cu^{2+}	Zn^{2+}	Ag^{+}	Cd^{2+}	Pb^{2+}	Hg^{2+}	%Class.
**H_2O**	9	0	0	0	0	0	0	0	0	0	0	1	0	90
**Ba^{2+}**	0	7	0	3	0	0	0	0	0	0	0	0	0	70
**Al^{3+}**	0	1	9	0	0	0	0	0	0	0	0	0	0	90
**Cr^{3+}**	0	1	0	8	0	0	0	0	0	0	0	1	0	80
**Fe^{2+}**	0	0	0	0	10	0	0	0	0	0	0	0	0	100
**Co^{2+}**	0	0	0	0	0	10	0	0	0	0	0	0	0	100
**Ni^{2+}**	0	0	0	0	0	0	10	0	0	0	0	0	0	100
**Cu^{2+}**	0	0	0	0	0	0	0	10	0	0	0	0	0	100
**Zn^{2+}**	0	0	0	0	0	0	0	0	10	0	0	0	0	100
**Ag^{+}**	0	0	0	0	0	0	0	0	0	10	0	0	0	100
**Cd^{2+}**	0	0	0	0	0	0	0	0	0	0	10	0	0	100
**Pb^{2+}**	0	0	0	0	0	0	0	0	0	0	0	10	0	100
**Hg^{2+}**	0	0	0	0	0	0	0	0	0	0	0	0	10	100

(%class. = %classifications)

Table S3. Numerical data of graphs shown in Figure 4.

Single spotting			10-time spotting		
$[Zn^{2+}]$ (μM)	Difference of Mean Gray Value	SEM	$[Zn^{2+}]$ (μM)	Difference of Mean Gray Value	SEM
0.20	6.21	0.78	0.00	5.91	2.41
0.40	10.93	1.54	0.01	5.96	2.44
0.60	13.89	2.26	0.02	7.53	3.07
0.80	19.53	2.46	0.04	6.99	2.85
1.00	23.10	1.94	0.06	7.42	3.03
2.00	36.83	1.61	0.08	7.36	3.01
4.00	51.27	3.08	0.10	7.35	3.00
6.00	65.94	7.00	0.20	7.14	2.91
8.00	71.82	5.10	0.30	7.41	3.02
10.00	78.54	4.32	0.40	7.98	3.26
20.00	106.55	2.37	0.50	7.87	3.21

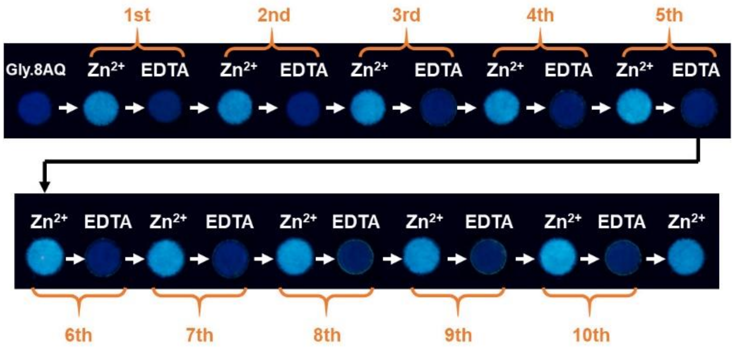

Figure S4. Fluorescence responses of **Gly.8AQ** in sensing Zn^{2+} after ten times. The sensor was recovered by sequestering Zn^{2+} with EDTA, a known chelator for Zn^{2+}.

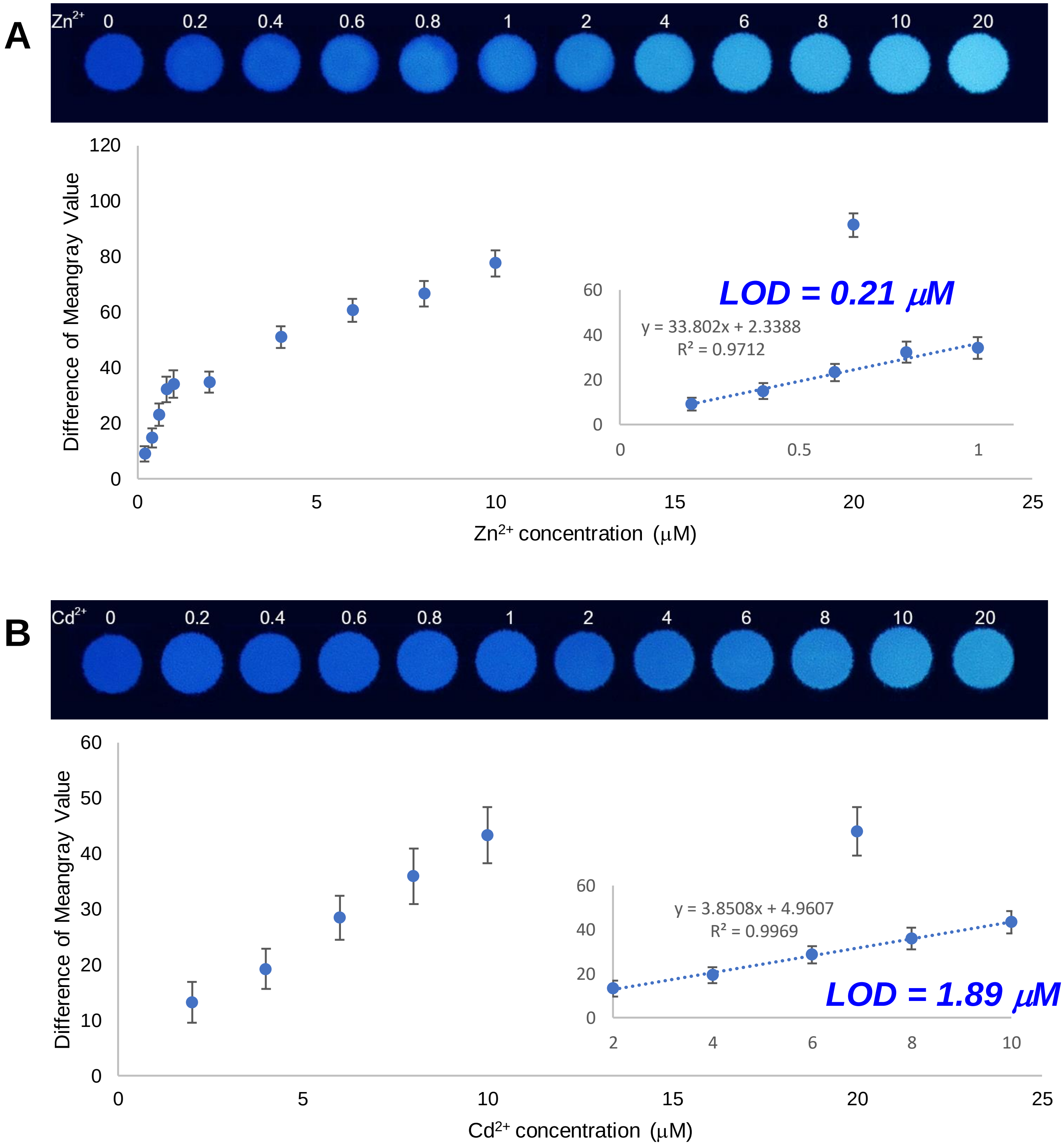

Figure S5. Response curves for **Asn.8AQ** in sensing (A) Zn^{2+} and (B) Cd^{2+}.

Fluorescence and UV-vis titrations

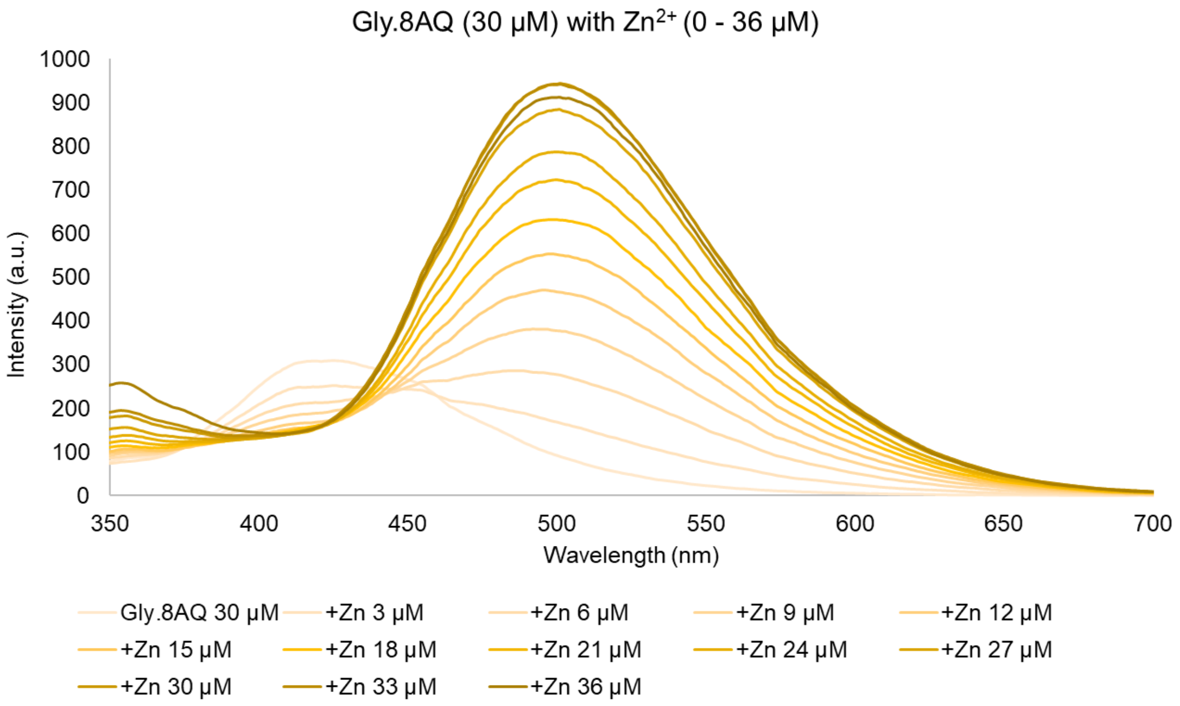

Figure S6. Fluorescence titration of 2-amino-*N*-(quinolin-8-yl)acetamide with Zn^{2+} (excited at 300 nm)

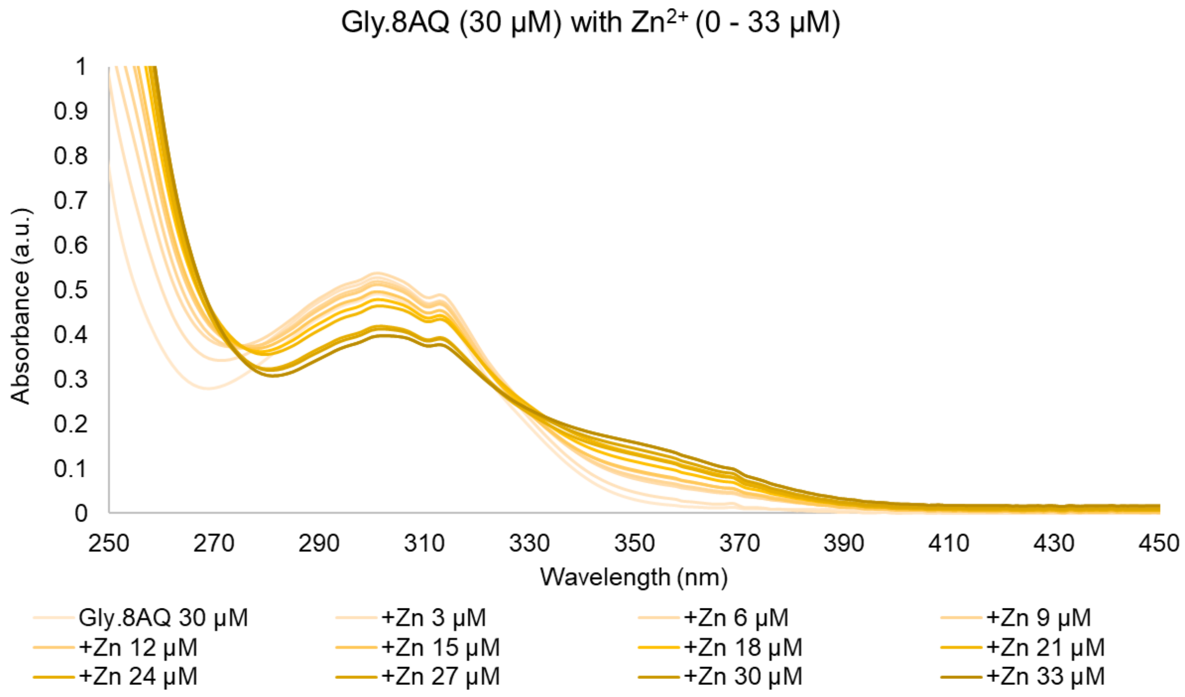

Figure S7. UV-vis titration of 2-amino-*N*-(quinolin-8-yl)acetamide with Zn^{2+}.

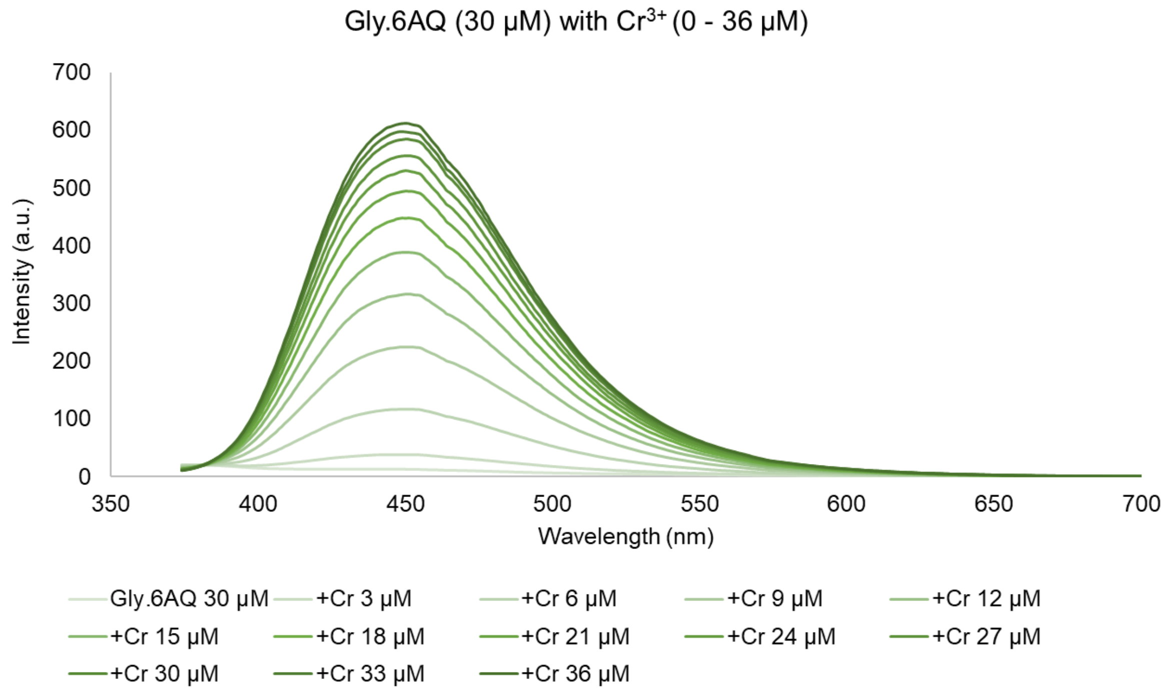

Figure S8. Fluorescence titration of 2-amino-*N*-(quinolin-6-yl)acetamide with Cr^{3+} (excited at 365 nm).

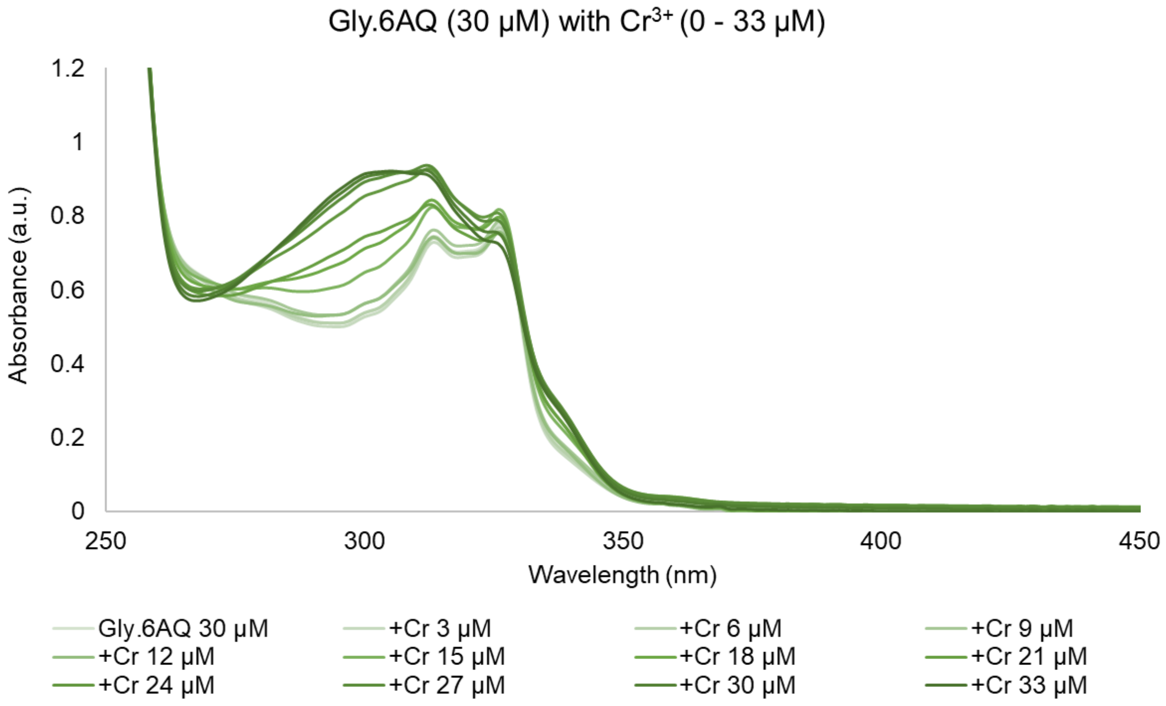

Figure S9. UV-vis titration of 2-amino-*N*-(quinolin-6-yl)acetamide with Cr^{3+}.

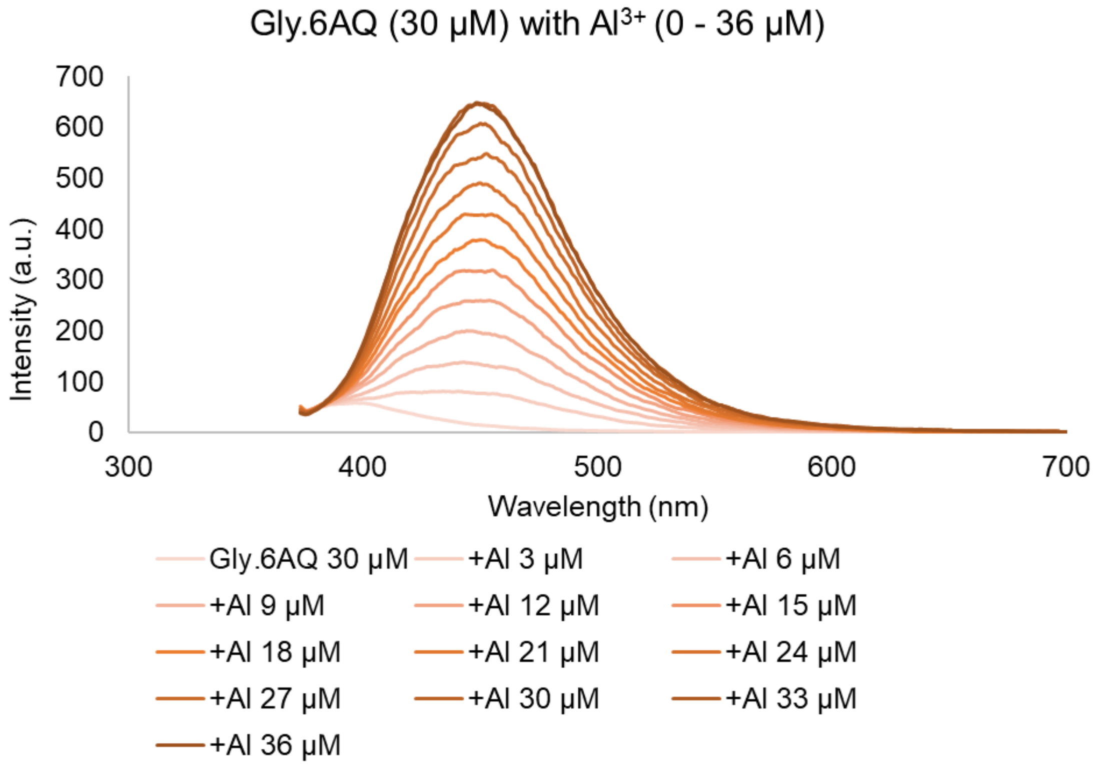

Figure S10. Fluorescence titration of 2-amino-*N*-(quinolin-6-yl)acetamide with Al^{3+} (excited at 365 nm).

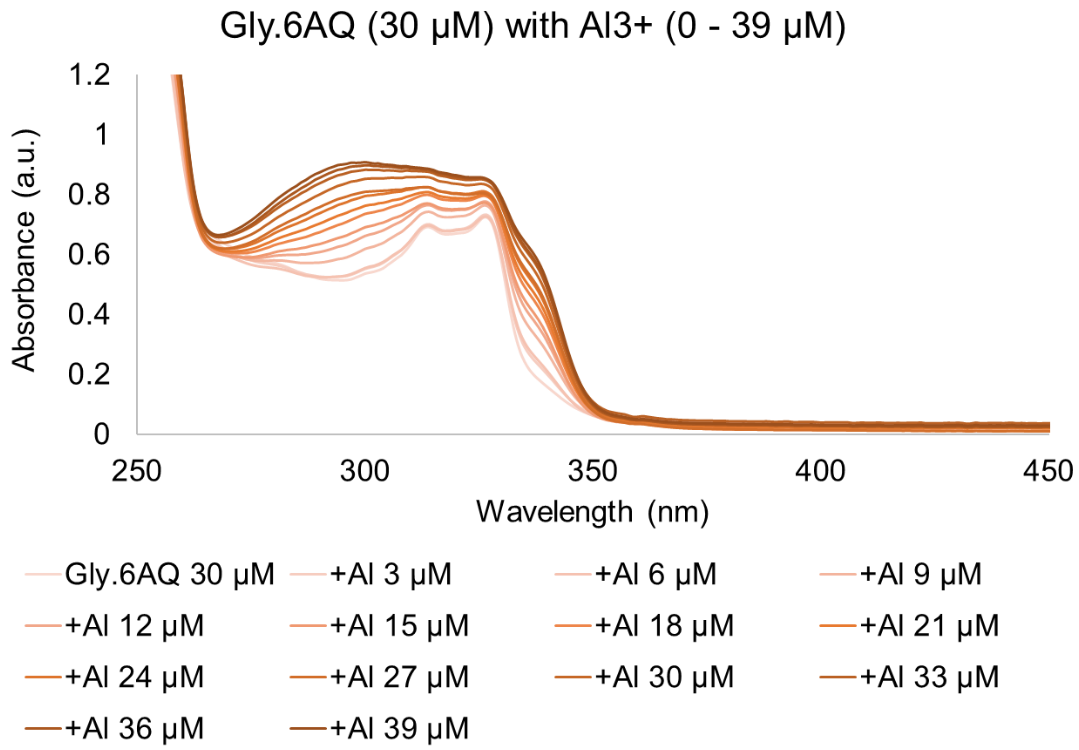

Figure S11. UV-vis titration of 2-amino-*N*-(quinolin-6-yl)acetamide with Al^{3+}.

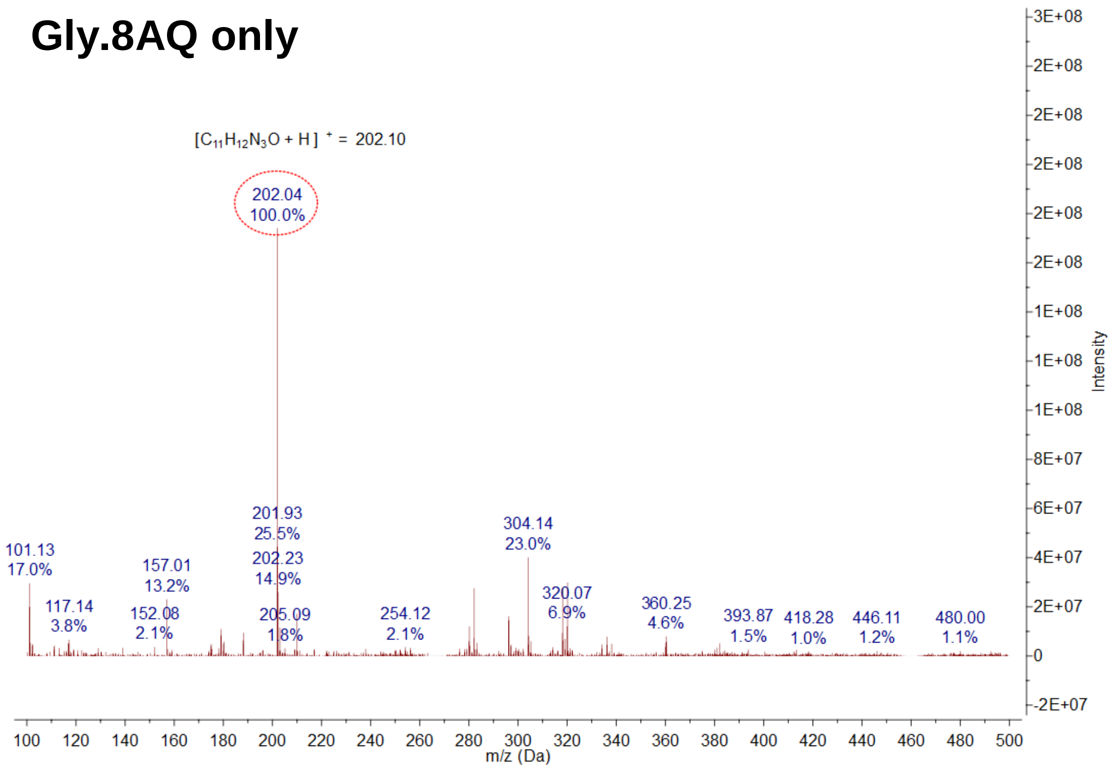
Gly.8AQ only
[C11H12N3O + H]+ = 202.10
202.04 100.0%
201.93 25.5%
202.23 14.9%
101.13 17.0%
117.14 3.8%
157.01 13.2%
152.08 2.1%
205.09 1.8%
254.12 2.1%
304.14 23.0%
320.07 6.9%
360.25 4.6%
393.87 1.5%
418.28 1.0%
446.11 1.2%
480.00 1.1%
Intensity
m/z (Da)

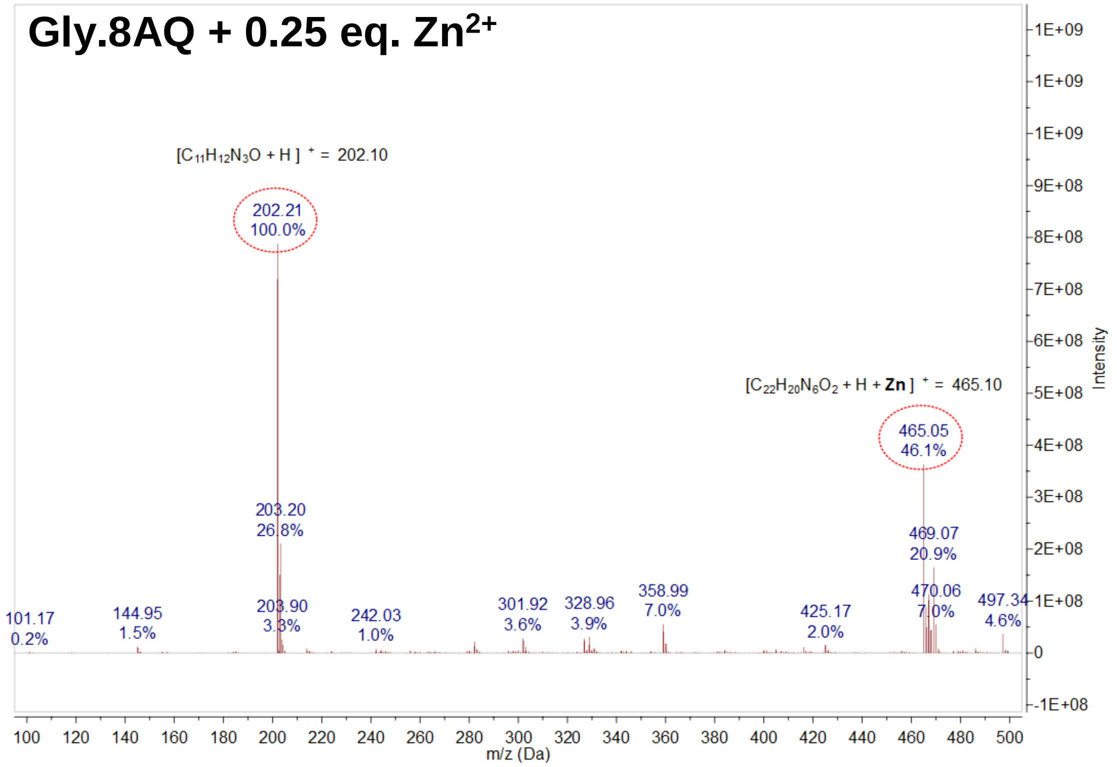
Gly.8AQ + 0.25 eq. Zn2+
[C11H12N3O + H]+ = 202.10
202.21 100.0%
203.20 26.8%
203.90 3.3%
101.17 0.2%
144.95 1.5%
242.03 1.0%
301.92 3.6%
328.96 3.9%
358.99 7.0%
425.17 2.0%
[C22H20N6O2 + H + Zn]+ = 465.10
465.05 46.1%
469.07 20.9%
470.06 7.0%
497.34 4.6%
Intensity
m/z (Da)

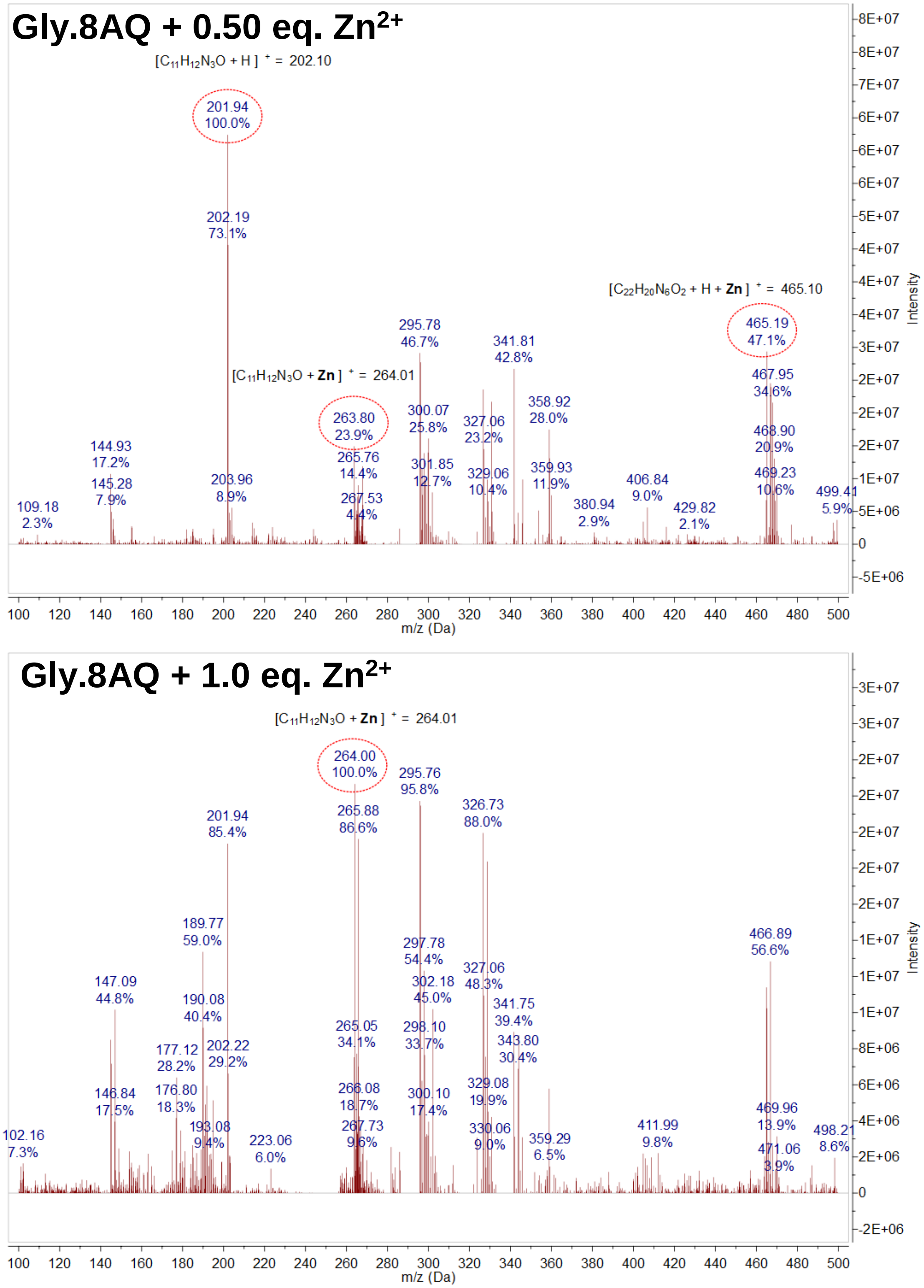

Figure S12. MS titration between surface-free **Gly.8AQ** and Zn^{2+}.

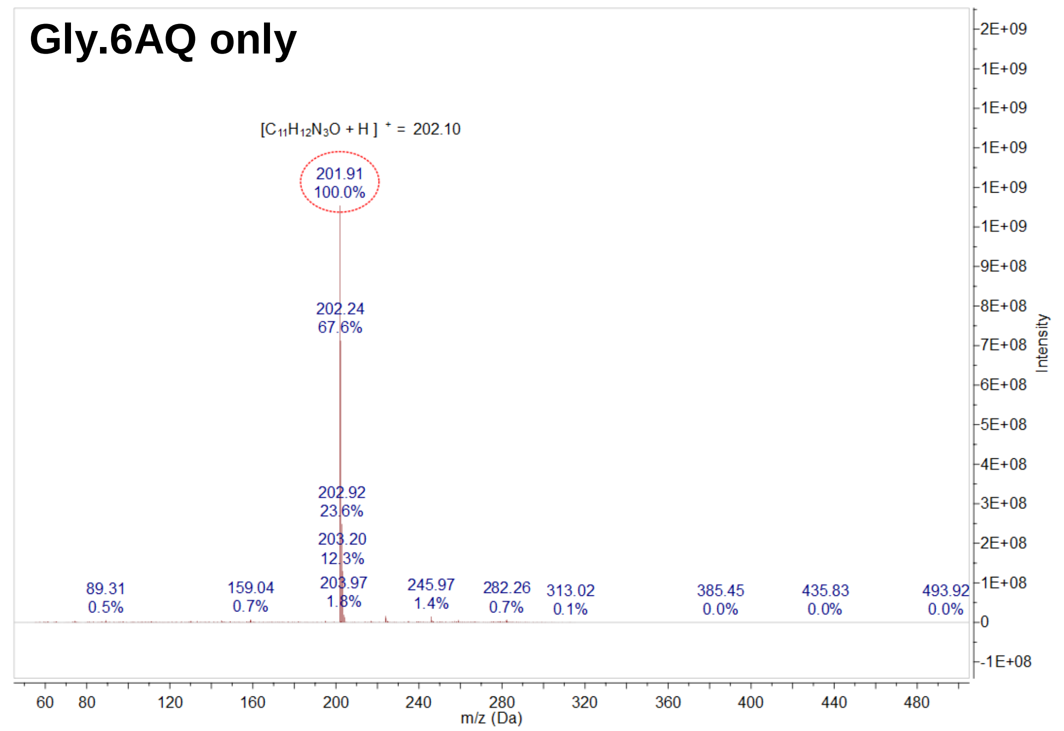

Gly.6AQ only
[C11H12N3O + H]+ = 202.10
201.91
100.0%
202.24
67.6%
202.92
23.6%
203.20
12.3%
89.31
0.5%
159.04
0.7%
203.97
1.8%
245.97
1.4%
282.26
0.7%
313.02
0.1%
385.45
0.0%
435.83
0.0%
493.92
0.0%
m/z (Da)
Intensity

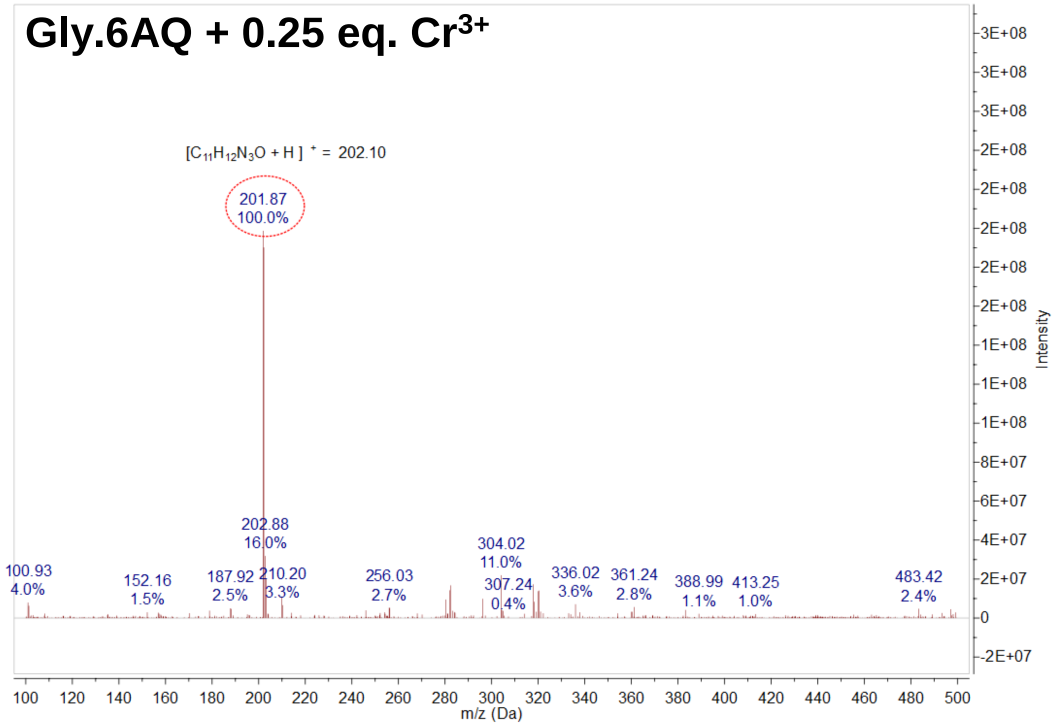

Gly.6AQ + 0.25 eq. Cr3+
[C11H12N3O + H]+ = 202.10
201.87
100.0%
202.88
16.0%
100.93
4.0%
152.16
1.5%
187.92
2.5%
210.20
3.3%
256.03
2.7%
304.02
11.0%
307.24
0.4%
336.02
3.6%
361.24
2.8%
388.99
1.1%
413.25
1.0%
483.42
2.4%
m/z (Da)
Intensity

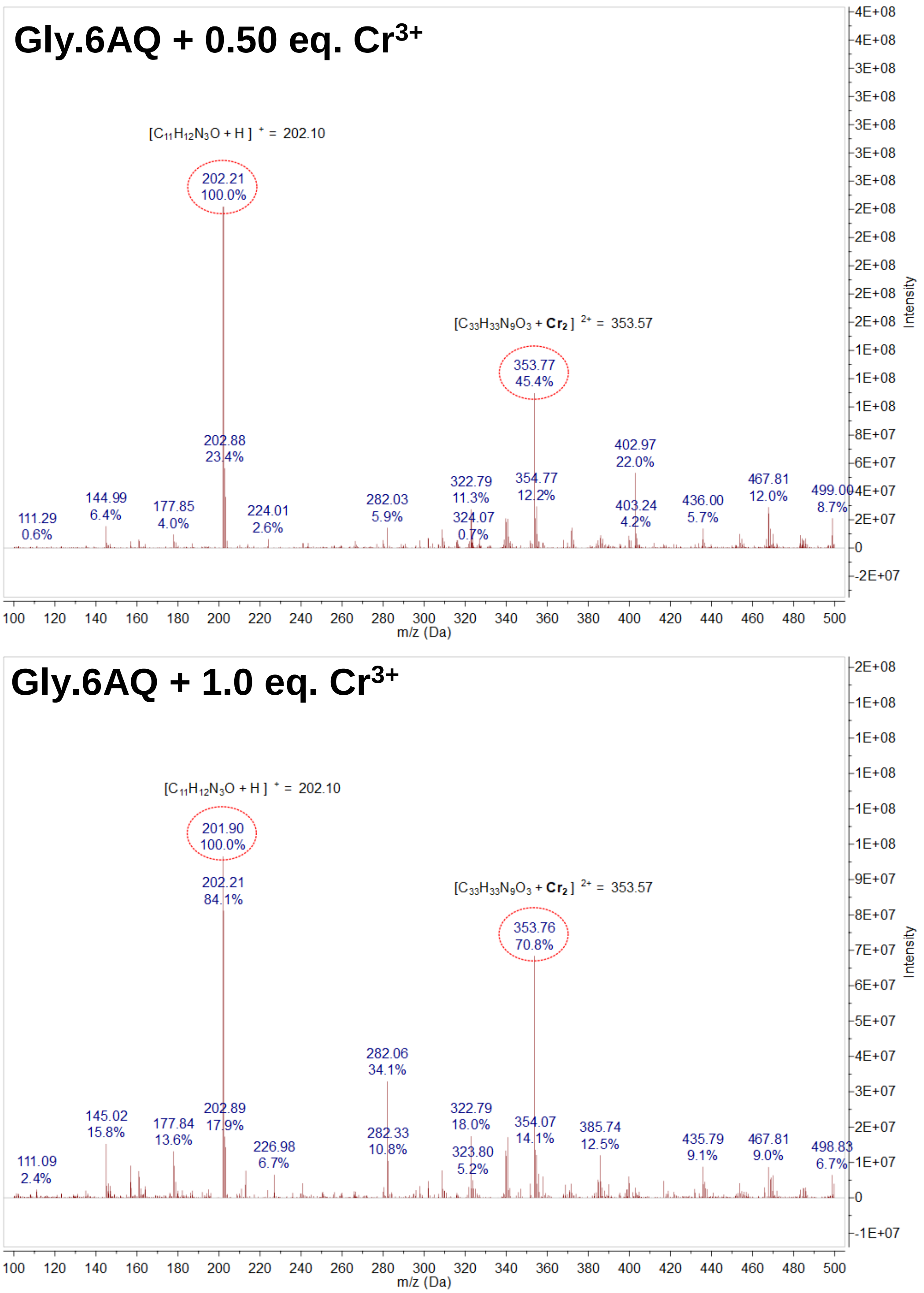

Figure S13. MS titration between surface-free **Gly.6AQ** and Cr^{3+}.

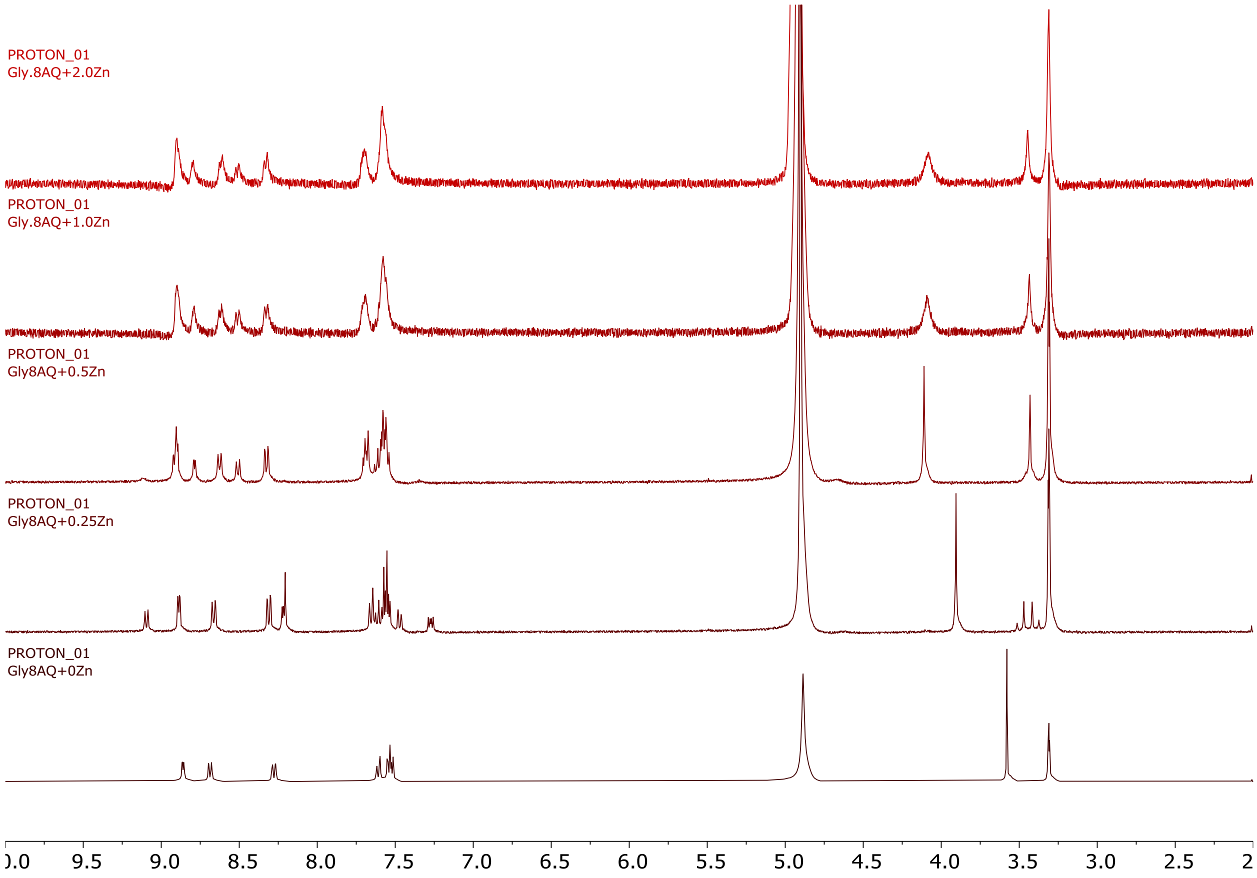

Figure S14. NMR titration between surface-free **Gly.8AQ** and Zn^{2+}.

Spectra data of representative fluorophores

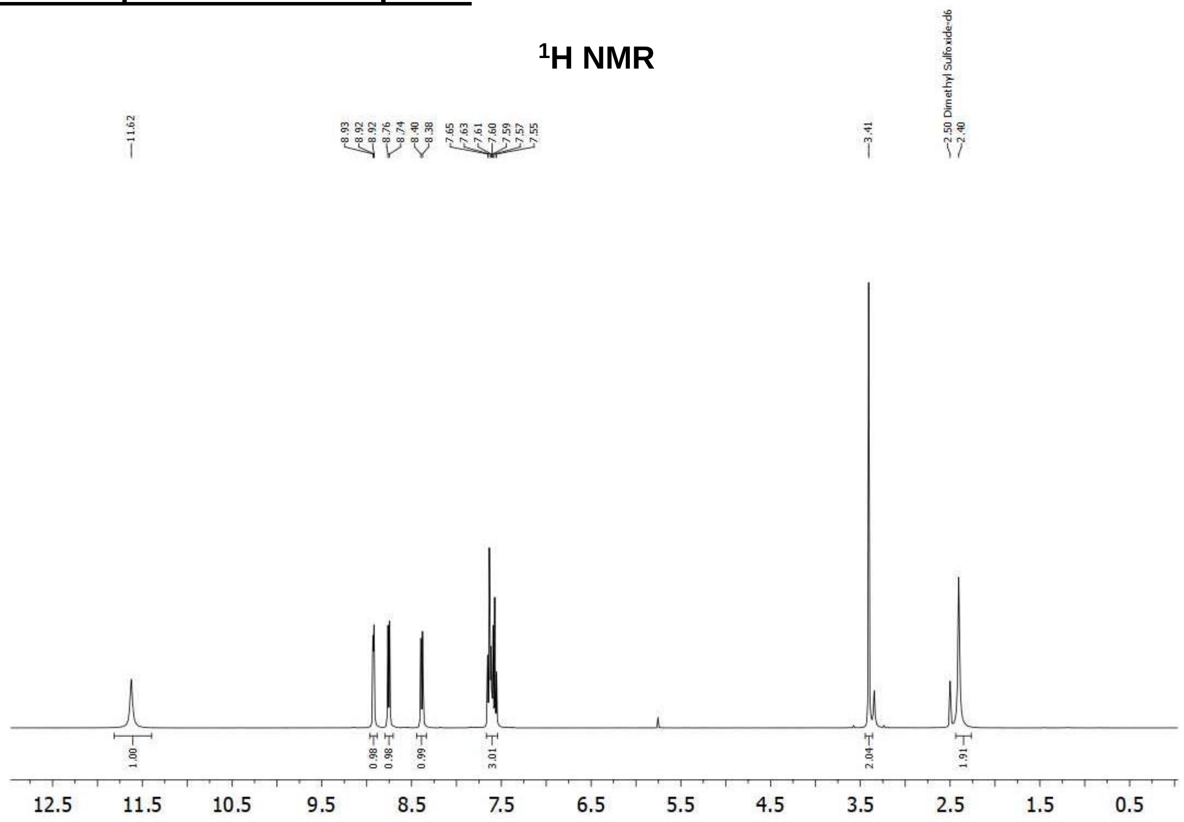

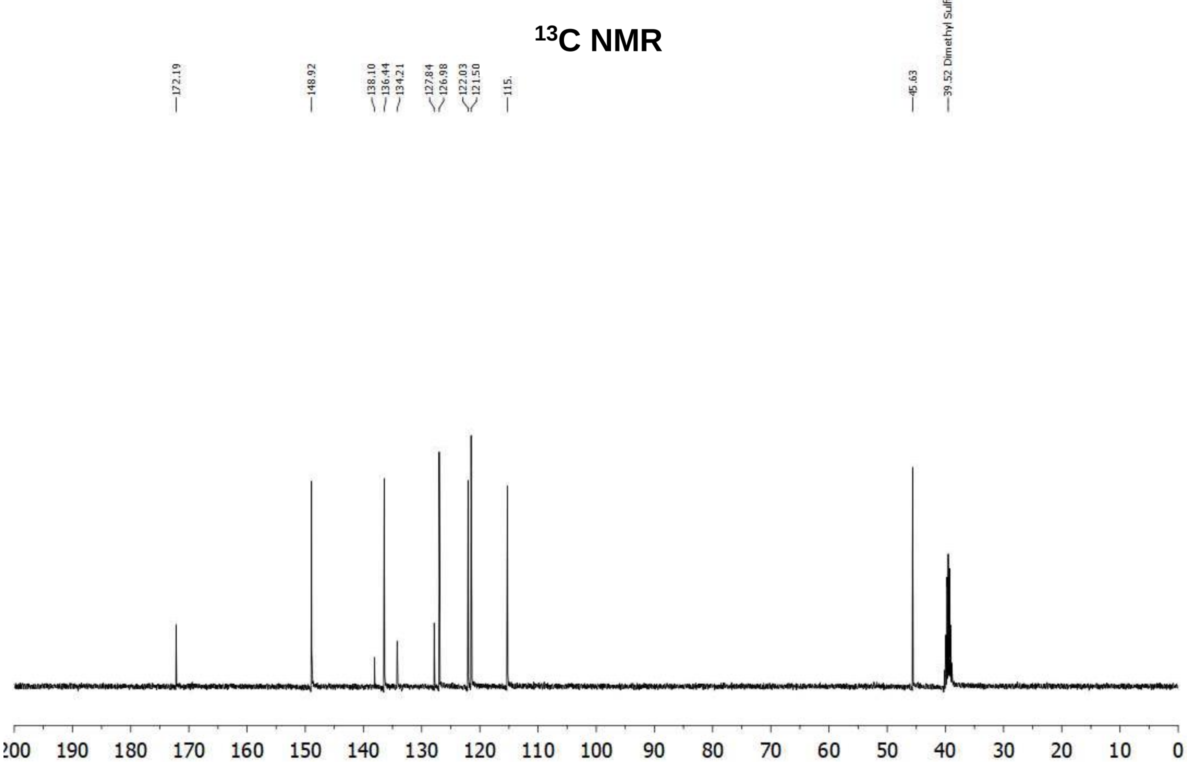

Figure S15. ^{1}H and ^{13}C NMR spectra of (2-amino-*N*-(quinolin-8-yl)acetamide) (unbound **Gly.8AQ**).

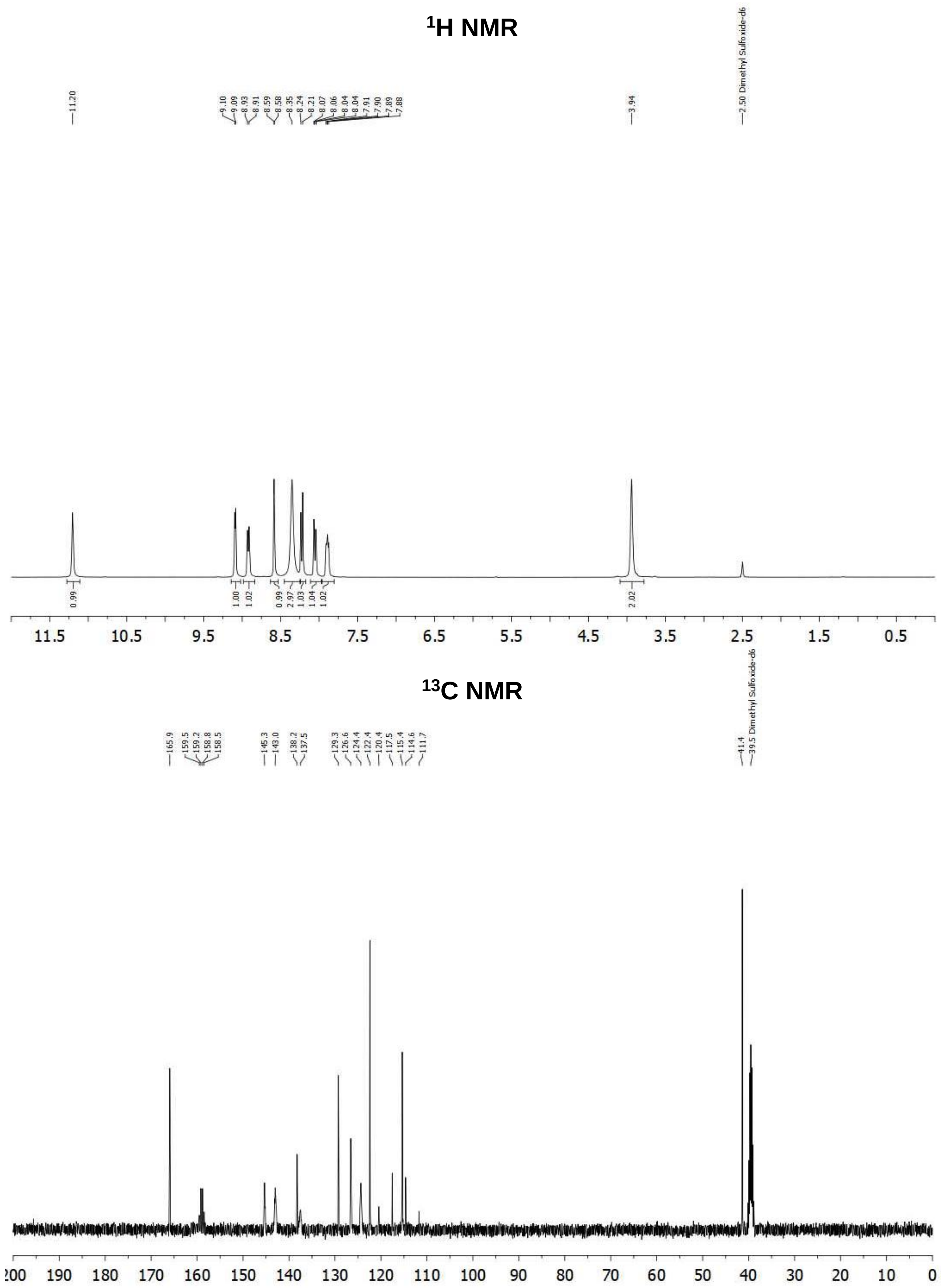

Figure S16. ^{1}H and ^{13}C NMR spectra of (2-amino-*N*-(quinolin-6-yl)acetamide) (unbound, TFA salt of **Gly.6AQ**).

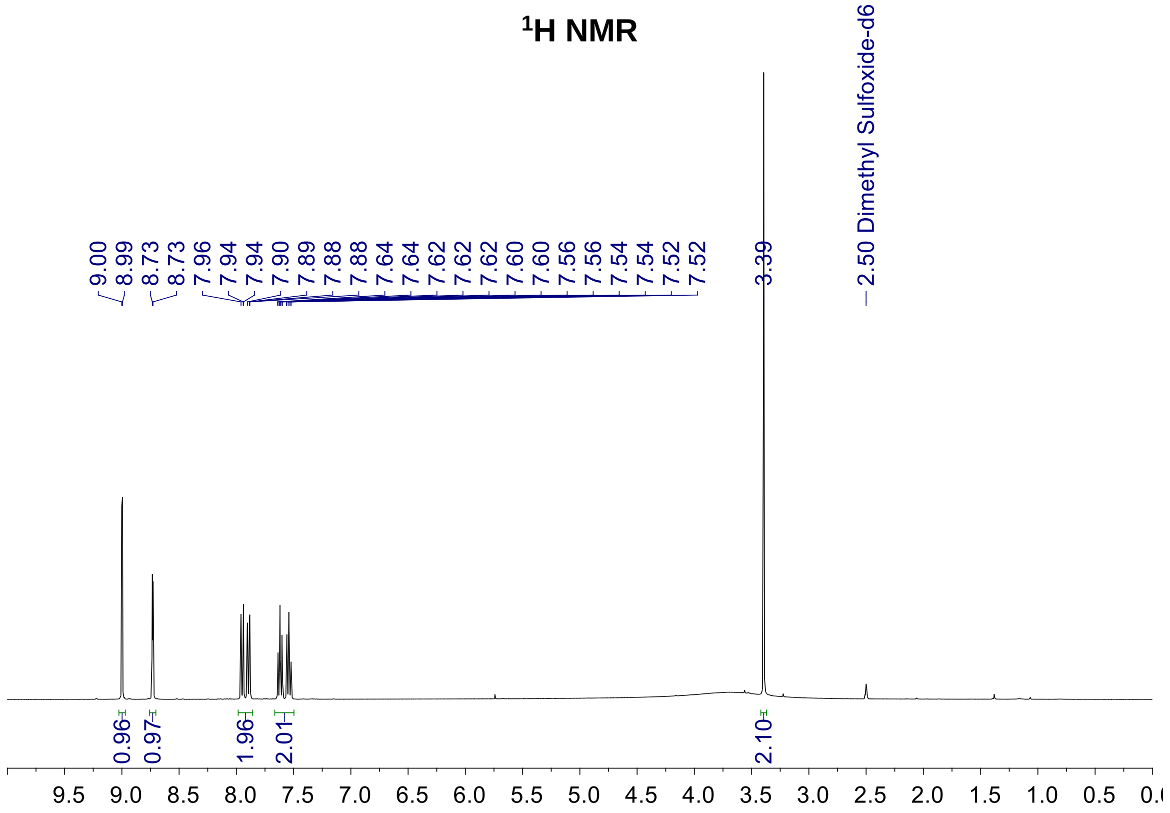

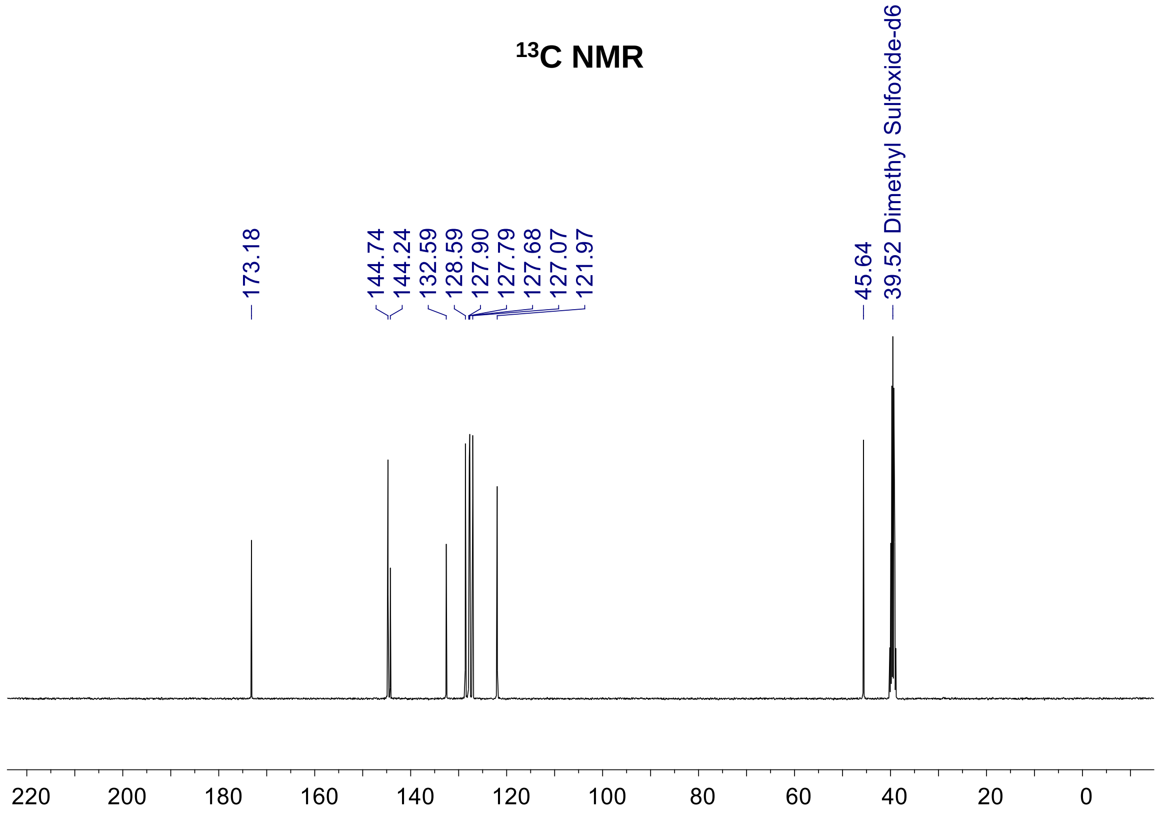

Figure S17. ^{1}H and ^{13}C NMR spectra of (2-amino-*N*-(quinolin-3-yl)acetamide) (unbound, TFA salt of **Gly.3AQ**).

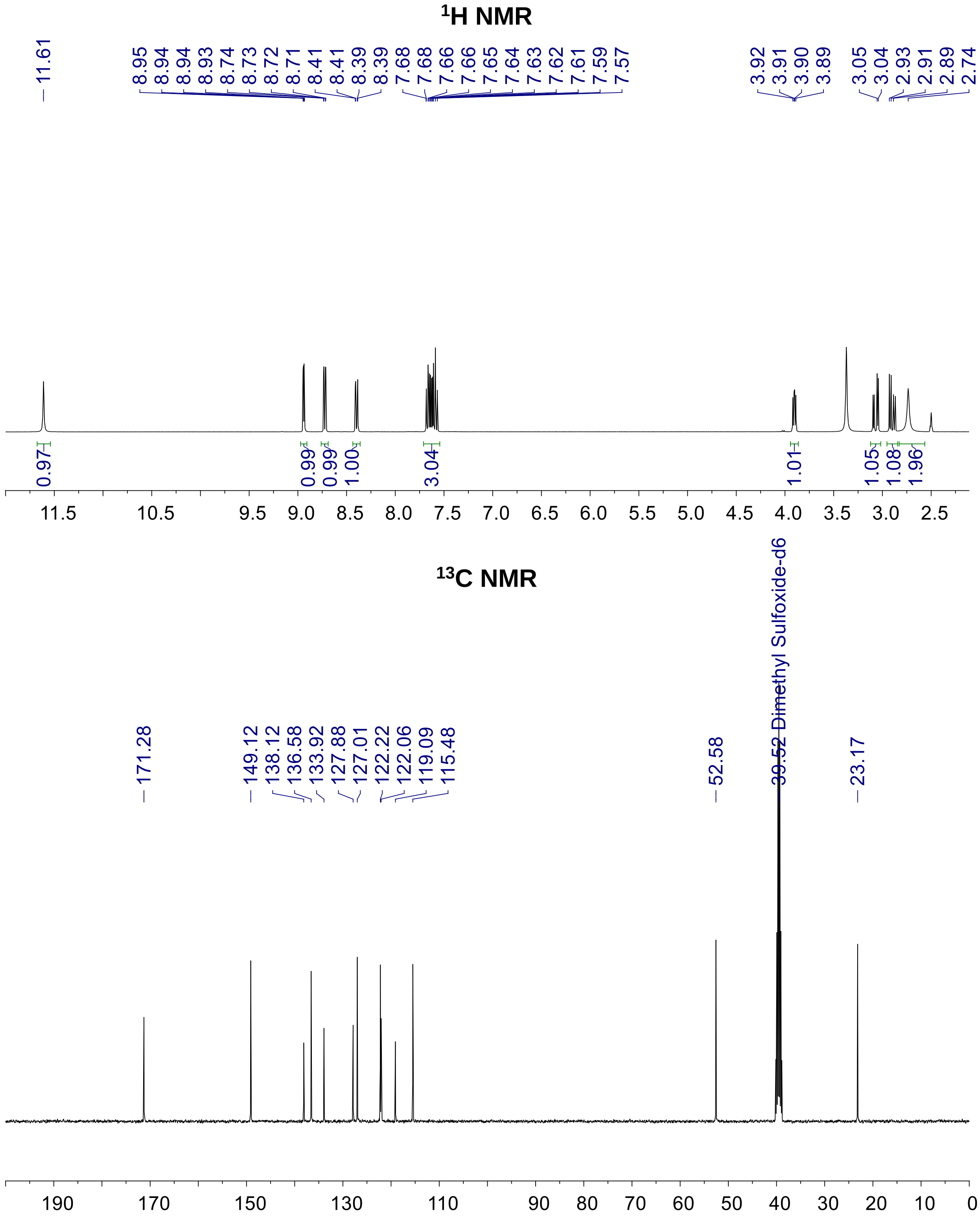

Figure S18. ^{1}H and ^{13}C NMR spectra of (2-amino-N^{1}-(quinolin-8-yl)succinamide) (unbound, TFA salt of **Asn.8AQ**).
